# A Metaproteomic Approach to Study Human-Microbial Ecosystems at the Mucosal Luminal Interface

**DOI:** 10.1371/journal.pone.0026542

**Published:** 2011-11-21

**Authors:** Xiaoxiao Li, James LeBlanc, Allison Truong, Ravi Vuthoori, Sharon S. Chen, Jonathan L. Lustgarten, Bennett Roth, Jeff Allard, Andrew Ippoliti, Laura L. Presley, James Borneman, William L. Bigbee, Vanathi Gopalakrishnan, Thomas G. Graeber, David Elashoff, Jonathan Braun, Lee Goodglick

**Affiliations:** 1 Department of Molecular and Medical Pharmacology, David Geffen School of Medicine at University of California Los Angeles, Los Angeles, California, United States of America; 2 Department of Pathology and Laboratory Medicine, David Geffen School of Medicine at University of California Los Angeles, Los Angeles, California, United States of America; 3 Department of Biomedical Informatics, University of Pittsburgh, Pittsburgh, Pennsylvania, United States of America; 4 Department of Medicine, Division of Digestive Disease, David Geffen School of Medicine at University of California Los Angeles, Los Angeles, California, United States of America; 5 Inflammatory Bowel Disease Center, Cedars-Sinai Medical Center, Los Angeles, California, United States of America; 6 Department of Plant Pathology and Microbiology, University of California Riverside, Riverside, California, United States of America; 7 Department of Pathology, University of Pittsburgh School of Medicine, Pittsburgh, Pennsylvania, United States of America; 8 University of Pittsburgh Cancer Institute, University of Pittsburgh, Pittsburgh, Pennsylvania, United States of America; 9 Department of Computational and Systems Biology, University of Pittsburgh School of Medicine, Pittsburgh, Pennsylvania, United States of America; 10 Jonsson Comprehensive Cancer Center, David Geffen School of Medicine at University of California Los Angeles, Los Angeles, California, United States of America; 11 Department of Medicine, David Geffen School of Medicine at University of California Los Angeles, Los Angeles, California, United States of America; 12 Department of Biostatistics, David Geffen School of Medicine at University of California Los Angeles, Los Angeles, California, United States of America; McGill University, Canada

## Abstract

Aberrant interactions between the host and the intestinal bacteria are thought to contribute to the pathogenesis of many digestive diseases. However, studying the complex ecosystem at the human mucosal-luminal interface (MLI) is challenging and requires an integrative systems biology approach. Therefore, we developed a novel method integrating lavage sampling of the human mucosal surface, high-throughput proteomics, and a unique suite of bioinformatic and statistical analyses. Shotgun proteomic analysis of secreted proteins recovered from the MLI confirmed the presence of both human and bacterial components. To profile the MLI metaproteome, we collected 205 mucosal lavage samples from 38 healthy subjects, and subjected them to high-throughput proteomics. The spectral data were subjected to a rigorous data processing pipeline to optimize suitability for quantitation and analysis, and then were evaluated using a set of biostatistical tools. Compared to the mucosal transcriptome, the MLI metaproteome was enriched for extracellular proteins involved in response to stimulus and immune system processes. Analysis of the metaproteome revealed significant individual-related as well as anatomic region-related (biogeographic) features. Quantitative shotgun proteomics established the identity and confirmed the biogeographic association of 49 proteins (including 3 functional protein networks) demarcating the proximal and distal colon. This robust and integrated proteomic approach is thus effective for identifying functional features of the human mucosal ecosystem, and a fresh understanding of the basic biology and disease processes at the MLI.

## Introduction

The intestinal mucosal surface plays diverse and critical roles in nutrient uptake, host defense, and local and systemic endocrinology [1], [2], [3], [4]. Anatomic regions of the intestine differ in these properties and their resultant disease susceptibility, in part due to corresponding differences in the composition and function of mucosal cell types. However, the functional state of the mucosa in health and disease is also profoundly affected by its interplay with luminal intestinal microbiota [5], [6]. Molecular phylotypic analysis has uncovered great complexity and inter-individual heterogeneity of the organisms comprising the intestinal microbiome [7], [8], [9]. Accordingly, functional assessment of the microbiome has recently emphasized metagenomic and biochemical analysis, uncovering commonalities in metabolic and other traits responsive to diet, and reciprocal interactions with host physiology [10], [11], [12], [13].

Thus far, such studies have almost exclusively focused on the biology of the fecal compartment, and there have been only limited assessments of the mucosal surface itself. Gnotobiotic mice have delineated extensive, reciprocal adaptive gene expression and functional change in both colonic mucosal and microbial populations [14], [15], and extensive and unique microbial communities have been detected at the human mucosal-luminal interface (MLI) [7], [8]. However, there are few strategies available to directly analyze the function of the MLI in humans as an integrated host-commensal biologic unit.

**Figure 1:**
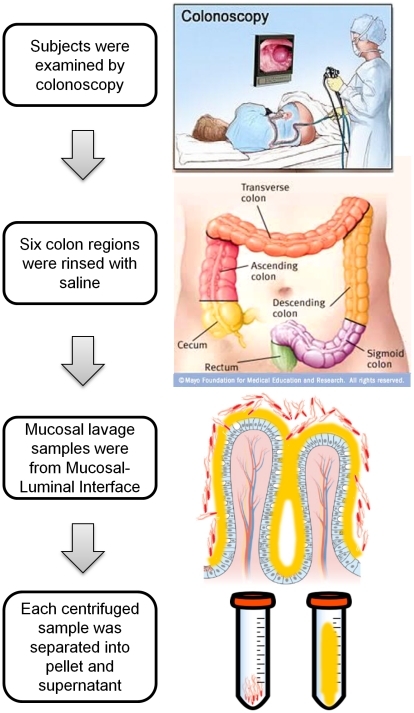
Flowchart of mucosal lavage sampling.

In this study, we introduced a novel approach to directly sample the MLI by endoscopic saline lavage of the mucosal surface at multiple anatomic regions in individual patients. High-throughput proteomic analysis and a suite of bioinformatics tools were utilized to profile the human mucosal metaproteome represented in these samples. A schematic flowchart of sample collection and initial characterization is illustrated in Figure 1. Using this approach, we found that mucosal lavage specimens contain components of both human and microbial origin. Comparison of inter- and intra-subject variation of the mucosal metaproteome found that a significant feature of the metaproteome is biogeographic, distinguishing the distal and proximal colon regions. We used quantitative shotgun proteomics to further identify host proteins carrying strong biogeographic features. The results showed that this new integrated sampling and analytical approach is capable of analyzing molecular compositions at different locations along the gastrointestinal tract, and hence provides a new dimension to the characterization of host-microbial interaction at the human MLI.

## Results

### Characterizing the phylotypic origin of mucosal lavage proteins

**Figure 2:**
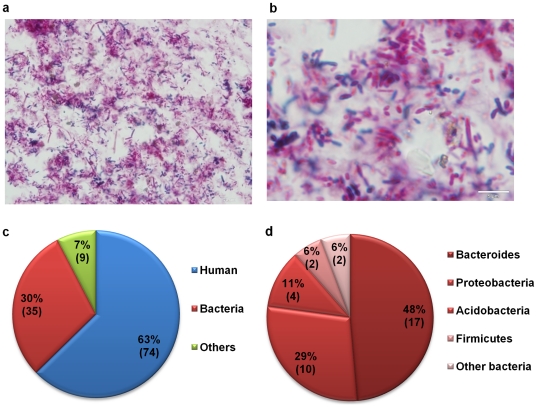
Cellular and protein composition at the MLI. Upper panel: Cytology analysis of the cell pellet obtained from each mucosal lavage sample using gram staining. a.100× b. 500×. Lower panel: Distribution of proteins with different origins identified from the mucosal lavage sample using shotgun proteomic analysis. c. Composition of proteins from all species as identified by tandem MS. Other origin includes phage and amoebozoa. d. Composition of bacterial proteins. Other bacterial origin includes *Chlorobi* and *Cyanobacteria.*

To study the mucosal luminal interface, we established a novel protocol to directly examine this environment using samples obtained by endoscopic lavage. Briefly, 30 ml of sterile saline was injected onto a discrete surface of the mucosal surface in each colon region. The wash was then collected by vacuum suction. Based on the live time imaging from the video camera attached to the colonoscope, approximately 1 cm^2^ of the mucosal surface area was thoroughly rinsed in each lavage procedure. We initially collected 18 mucosal lavage samples from 6 intestinal regions of 3 healthy individuals undergoing cancer surveillance colonoscopic screening. Samples were centrifuged to separate the insoluble components (pellet) from the supernatant. We first analyzed the 18 cell pellets by cytology with Gram (Fig. 2a, b) or hematoxylin and eosin (H&E) staining. We observed 99% of the pellet consisted of bacterial cells. Human cell or food debris was only rarely observed. The bacteria population was a mixed population, consisting of both Gram-negative and -positive organisms with diverse morphological features.

To get a general estimate of the soluble composition of the MLI, supernatant fractions from the 18 mucosal lavage fluid specimens were combined, processed, trypsin digested, and analyzed by a shotgun proteomic approach. We were able to characterize tryptic peptides from 117 unique proteins in the sample, among which 63% (74) were human proteins, 30% (35) were bacterial, and 9 proteins were from phage or Amoebozoa (Fig. 2c). A list of identified proteins is provided (Table S1). Among the bacterial proteins, 48% belonged to the *Bacteroidetes,* the most abundant phylum of human intestinal microbiota. We also observed proteins from other bacterial phyla, including *Proteobacteria*, *Acidobacteria*, *Firmicutes*, *Chlorobi*, and *Cyanobacteria* (Fig. 2d). These findings demonstrated that mucosal lavage proteins represent a mixture of host and microbial products.

### Profiling the mucosal metaproteome in healthy subjects

To establish a more detailed characterization of the human mucosal metaproteome, we collected an additional 205 lavage samples from 38 healthy individuals. Typically, samples from six different colon regions were collected from the same subject. The demographics of the study population are summarized in Table 1. Each lavage sample was pre-processed and analyzed identically by matrix-assisted laser desorption/ionization (MALDI) time-of-flight (TOF) mass spectrometry (MS) proteomics in duplicates or triplicates. In total, 491 MALDI-TOF-MS spectra were collected from the 205 samples.

**Table 1:** Summary of sample collection and clinical traits.

**Total subjects**		38
**Total mucosal lavage samples**		205
**Gender**	**Female**	84 (41%)
	**Male**	121 (59%)
**Age**	**Median ± SD**	59±10
**Region**	**Cecum**	37 (18.0%)
	**Ascending**	35 (17.1%)
	**Transverse**	34 (16.6%)
	**Descending**	38 (18.5%)
	**Sigmoid**	31 (15.1%)
	**Rectum**	30 (14.6%)

**Figure 3:**
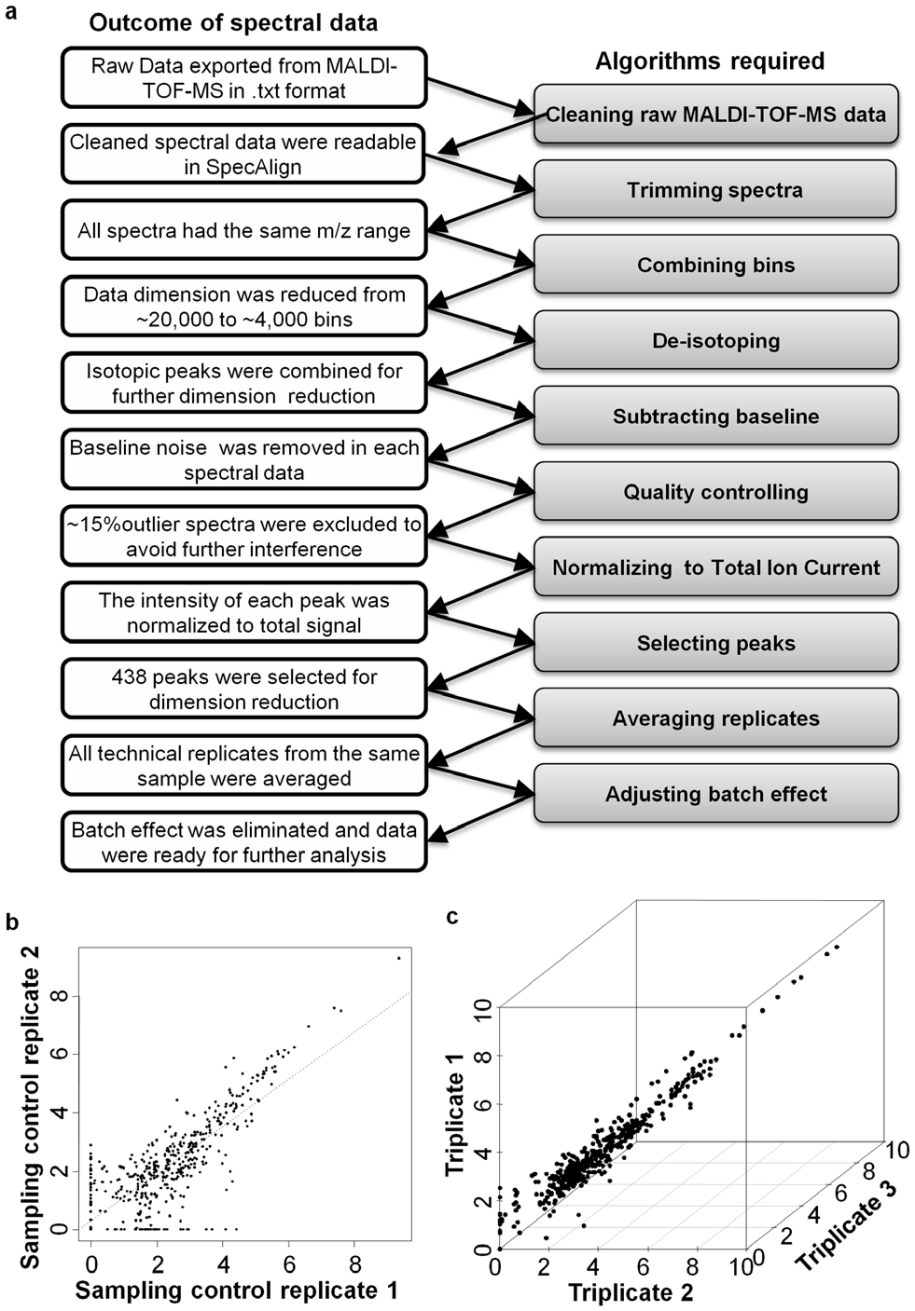
Reproducibility of metaproteomic analysis. a. Steps for MALDI-TOF-MS data pre-processing. b. Scatterplot of two samples obtained from the sample subject in adjacent colonic regions (sampling replicates). c. A representative 3D scatterplot of triplicate runs of the same sample (technical replicates).

High-resolution, high-throughput mass spectrometric proteomics presents a variety of challenges in data pre-processing and analysis [16]. Also, there are few mature commercially-available data management platforms, nor widely-accepted standards, for comprehensive proteomics data management. Therefore, we established a working protocol for high-resolution MALDI-TOF-MS data management from inception to final analysis, using software that is freely downloadable and open-source. The detailed spectral pre-processing procedure is summarized in Figure 3a.

To process high-resolution MALDI data, we used a recent edition of SpecAlign [17]. After pre-processing, 438 protein/peptide features (“peaks”) were selected from each metaproteomic spectrum. Each peak was labeled using the m/z value detected by MALDI-TOF-MS. In addition, we developed a stringent quality control protocol to remove outlier spectra. Each spectrum was inspected individually at two levels: total ion intensity level and average correlation with the entire dataset. Spectra with both intensity level and average correlation in the bottom 20% of the spectral profiles were disqualified, yielding a rate of outliers of 1.2%. Six spectra from two samples failed quality control criteria, likely due to low protein concentration. The remaining 485 spectrum data from 203 samples were used for subsequent study.

Prior to analyzing the complete set of data, we set out to characterize the reproducibility and robustness of our strategy. First, to test the reproducibility of the mucosal lavage sampling strategy, we collected lavage samples from two adjacent mucosa sites in the transverse colon. The sampling control replicates showed similar spectrum and had a correlation of 0.97 (Figure 3b). This indicated that our sampling strategy was reproducible. To further assess the reproducibility of sample processing and instrumental analysis, we also calculated the correlation between duplicate or triplicate samples. The average correlation coefficient is 0.99, indicating a high reproducibility of sample processing (Fig. 3c).

Due to the large number of samples, it was necessary to divide analysis into several MALDI-TOF-MS runs. This could potentially introduce an instrumental batch-effect noise which would contribute an artificial variance to our final results [18]. Therefore, we not only randomized all the samples to 7 batches, but also included batch control samples in quadruplicates on every 96-well MALDI-target plate for batch effect assessment. Not surprisingly, correlation analysis revealed a batch effect, as the intra-batch average correlation was 0.99, higher than the inter-batch average correlation 0.96 (Table S2). Therefore, a subsequent step was included to remove the batch effect before further analysis [19]. To adjust for batch effects in nucleotide microarray data, the empirical Bayes framework has proven robust for both large and small sample size. We adopted this strategy and performed similar adjustment on our MALDI-TOF-MS spectral data. After correction, the batch effect was no longer a predominant source of variance in the data, thus allowing us to characterize biological variations and similarities.

### Determining individual and biogeographic-related features of metaproteome

**Figure 4:**
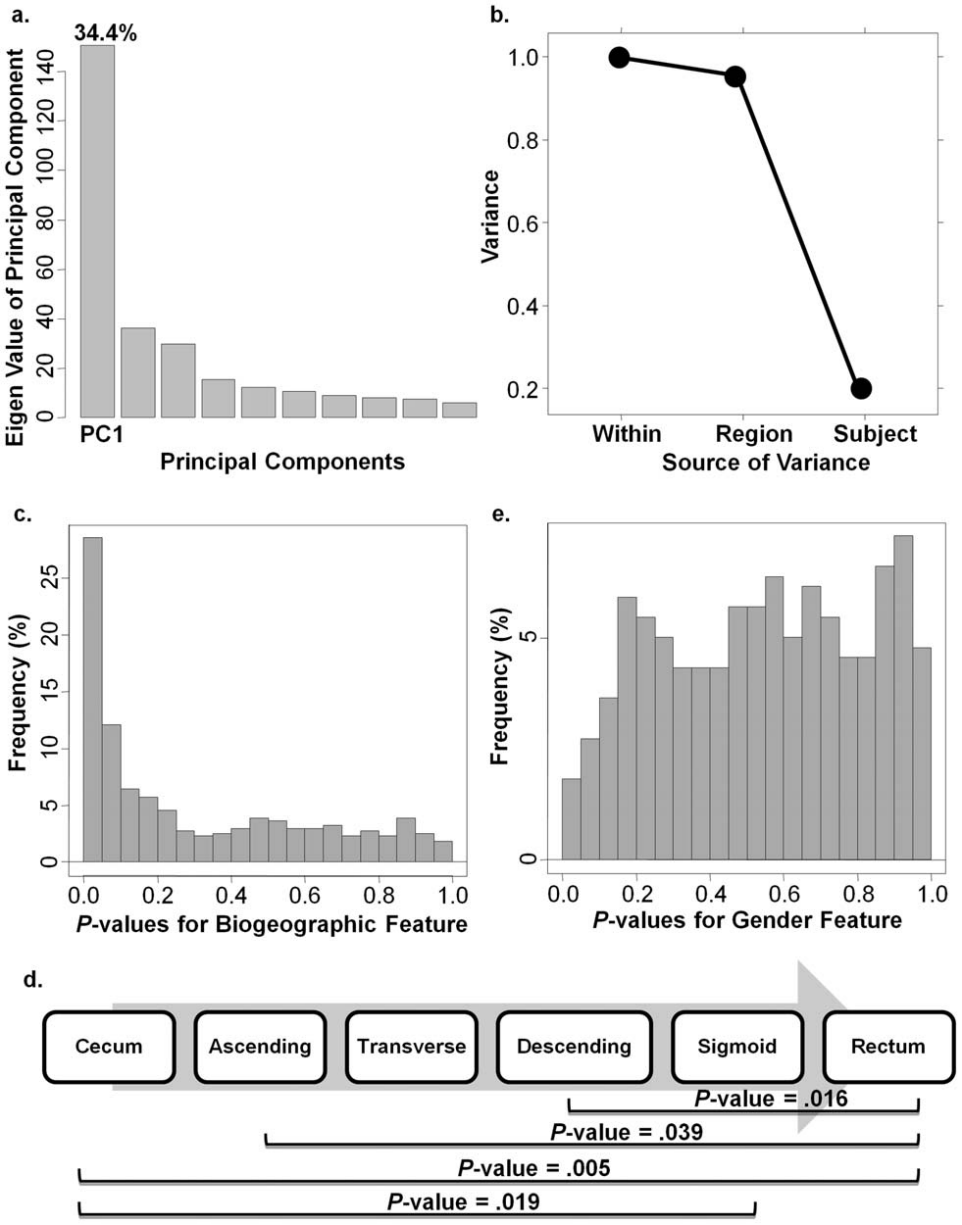
Features of human mucosal metaproteome. a. PCA analysis revealed PC1 represents the largest component of the overall variance. b. Variance component analysis showed variance in PC1 comes from both individual and biogeographic levels. c. Frequency plot of region-related *P*-value for each peak from NLME analysis indicated a significant biogeographic feature. d. Frequency plot of gender-related *P*-value for each peak from NLME analysis indicated no significance. e. Distal colon regions were significantly different from proximal regions in permutation analysis. Only significant *P*-values (<0.05) were shown.

After pre-processing, the metaproteome dataset containing 438 peaks from 203 samples was analyzed by a number of biostatistical methods (Dataset S1). First, to gain an overview of the source of variance in the human mucosal metaproteome, we conducted a principal variance component analysis. We first used a common multivariate method, Principal Component Analysis (PCA), to reduce the dimensions of the variance. This analysis revealed the first component (PC1) accounts for 34.4% of the total variance in the data (Fig. 4a). Second, we performed variance component analysis using a non-linear mixed-effect model (NLME) on PC1 specifically [20]. This analysis revealed that inter-subject (individual) factors contributed the majority of the variance. However, within each individual, there was a 5% biological variance among regions of the colon (referred to as ‘intra-subject, biogeographic factor’) (Fig. 4b). Consistent with other metagenomic data [7], the inter-subject difference was more predominant than the intra-subject level.

Next, we further examined the significance of the intra-subject biogeographic feature. Because of the hierarchical structure of the variation resources, we used the NLME model to compensate for individual factor. We analyzed the metaproteomic data to find peaks with differential abundance across the 6 colon anatomic regions. We then calculated the *P*-value which indicates the significance of the biogeographic feature for each peak (Table S3), and tabulated all 438 *P*-values on a frequency plot. If the effect was a random observation, less than 5% of the total peaks would have *P*-values smaller than 0.05. Instead, we observed a non-uniform distribution with over 25% of the peaks with *P*-values below 0.05 (Fig. 4c), suggesting the observed biogeographic feature was indeed significant.

To determine the similarities and differences among the 6 anatomic regions, we carried out a permutation test [21]. Only the significant *P*-values from this analysis were shown in Fig. 4d. No significant difference was observed in any adjacent two regions. However, we discovered significant differences between the proximal colon (including cecum, ascending, transverse, and descending colon), and the distal colon (including the sigmoid and rectum). Notably, the transverse colon (the most central colonic region) did not significantly differ with either proximal or distal colonic regions.

Lastly, we noticed our analysis left 20% of the PC1 unexplained by either individual or biogeographic effects. We speculated that it might be a mixed contribution from other physiological factors, such as age or gender. However, when tested in NLME or permutation test, neither age nor gender reached statistical significance, indicating that gender or age alone was not a significant factor in determining the mucosal metaproteome. The frequency plot of *P*-values for gender factor was shown as a negative example (Fig. 4e).

### Comparison of mucosal metaproteome and transcriptome

We next sought to biochemically validate the bioinformatically-defined biogeographic feature. To do so, we used a quantitative shotgun proteomic methodology [22] to directly identify proteins in sets of randomly selected individual samples from cecum, ascending colon, sigmoid, and rectum. As we expected, searches against different protein databases identified both microbial and human proteins. Here, we specifically focused on human proteins since they have the richest annotation information for subsequent analysis. In total, 31,224 spectra were collected and 300 human proteins were identified. The complete protein list and the spectrum count is organized into a spreadsheet (Dataset S2).

**Figure 5:**
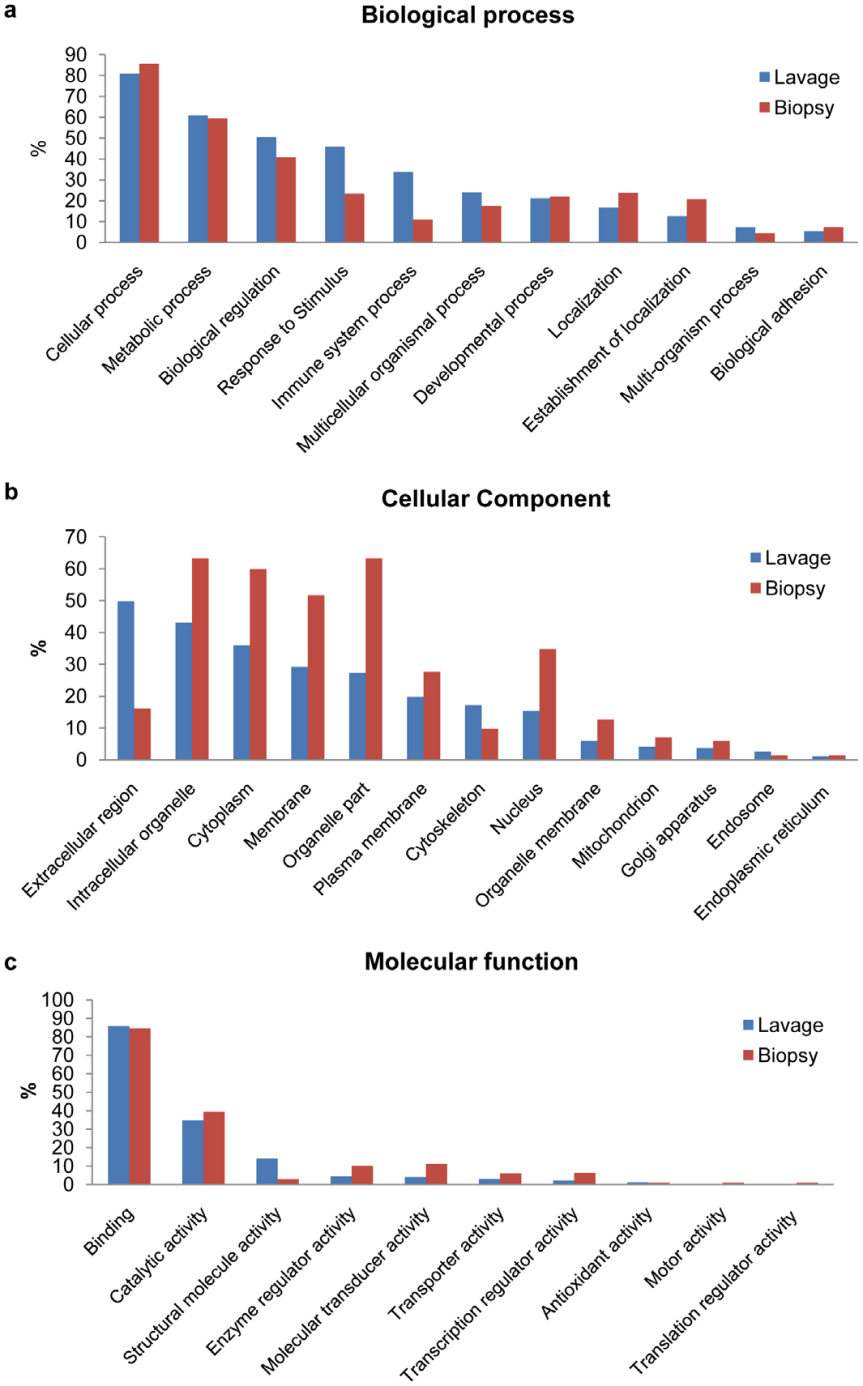
Comparison of lavage and biopsy samples by protein annotations. a. Biological process. b. Cellular component. c. Molecular function.

To determine the association of proteins isolated from colonic lavage compared to cells from biopsied tissues, we annotated the proteomic data in Scaffold using the latest protein information available from UniproKB/Swiss-Prot database. Since high dimensional proteomic datasets are not available from mucosal biopsy samples, we used for comparison the mucosal transcriptome recently reported as mRNA expression data for a large human mucosal biopsy sample set [23], and annotated using GeneTools [24]. Figure 5 shows the comparisons of the two datasets where the proteome software has defined expressed product based on “biological processes”, “cellular components”, and “molecular functions”. For biological process annotations, proteins involved in response to stimulus and immune system process were enriched in lavage samples compared to biopsies (Fig. 5a). For cellular components, the extracellular protein segment was greatly enriched in the lavage samples compared to biopsies as expected (Fig. 5b). For the segments defined by molecular function, the identified expression profile between the lavage samples and biopsies were similar with the majority of the proteins/transcripts involved in binding and catalytic activity (Fig. 5c).

### Identifying specific proteins with biogeographic feature

**Figure 6:**
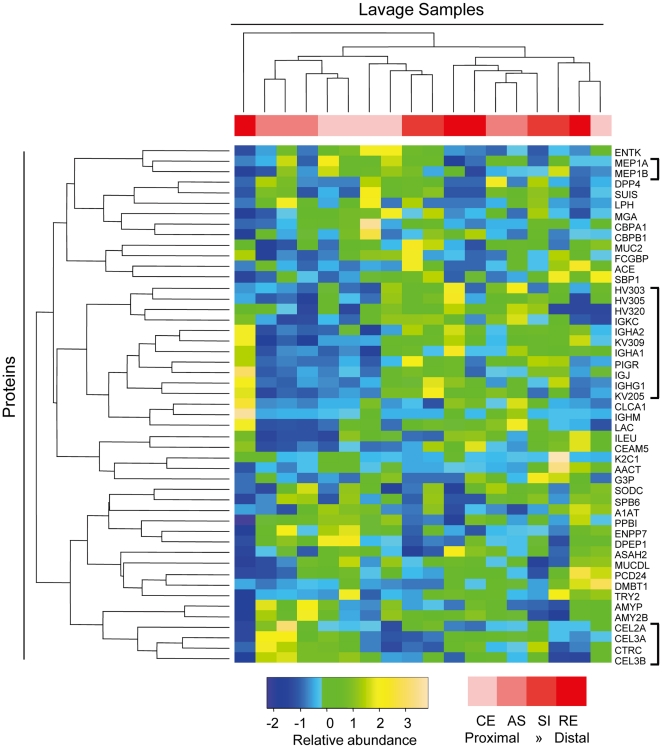
Heatmap view of proteins identified by shotgun proteomics. Horizontal tree indicates 18 independent lavage samples. Vertical tree indicates the 49 proteins analyzed, and the protein identities are listed on the right. Three of the proteins clusters showed biogeographic features are bracketed.

For the 300 human proteins identified from lavage samples, we used a stringent quality control protocol (described in Materials and Methods) to filter out proteins with poor coverage or low abundance. The ‘label-free spectrum counting’ method has been adopted to quantify the relative abundance of protein in analyzing shotgun proteomic data [22], [25], [26]. The Z-score transformed relative abundance of each protein is presented in a heatmap (Fig. 6), and the samples as well as the proteins were co-clustered by Pearson correlations (Fig. 6). The sample tree was mainly segregated into two large clusters with one outlier sample isolated to the far left. The first cluster consisted of 7 proximal and 2 sigmoid samples, whereas the second cluster consisted of 5 distal and 3 proximal samples. This suggested that the 49 proteins were differentially detected in proximal and distal regions, further confirming the biogeographic segmentation.

**Figure 7:**
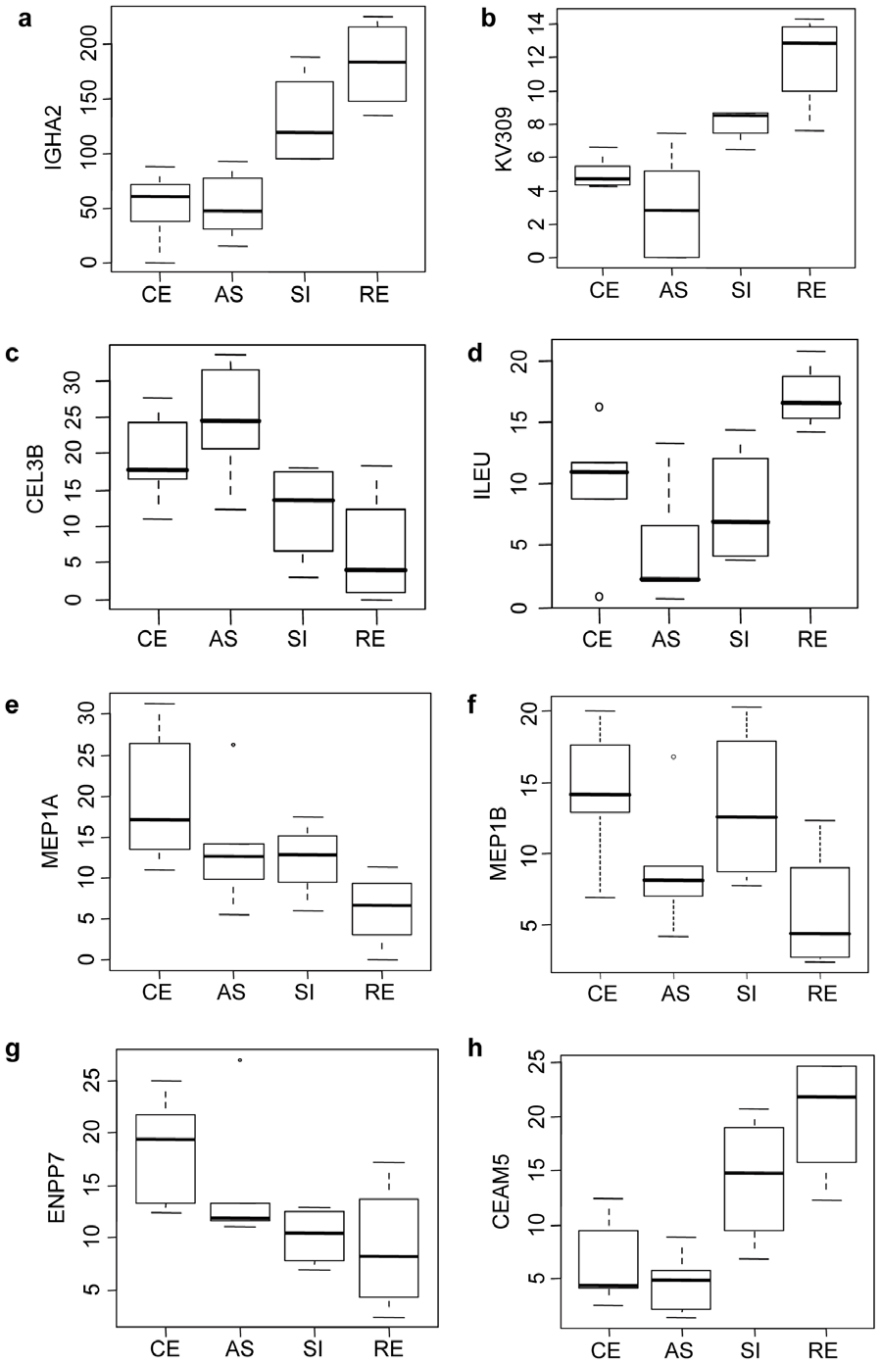
Boxplots of representative proteins with biogeographic features. The bottom and top of the boxes are the 25th and 75th percentile (the lower and upper quartiles, respectively), and the band near the middle of the box is the median. The whiskers present minimum to maximum value in the group. CE: cecum. AS: ascending colon. SI: sigmoid colon. RE: rectal colon. ANOVA is used for comparison between different regions. a. IGHA2 (P<0.001). b. KV309 (P<0.001). c. CEL3B (P = 0.017). d. ILEU (P = 0.017). e. MEP1A (P = 0.069). f. MEP1B (P = 0.044). g. ENPP7 (P = 0.014). h. CEAM5 (P<0.001).

As shown in Figure 6 (brackets on the right-side y-axis), there were a number of similar proteins that co-clustered. We selected several of these proteins for individual analysis with regard to biogeographical expression levels (Fig. 7). First, the immunoglobulin-related proteins Ig gamma-1 chain C region (IGHG1), Ig alpha-2 chain C region (IGHA2), Ig kappa chain V-III region VG (KV309), and polymeric immunoglobulin receptor (PIGR) all showed a similar pattern of lower expression in the proximal colon compared to the distal colon (Fig. 7a and b). This is consistent with the abundance of plasma cells and epithelial specialization for immunoglobulin transcytosis at this anatomic location. Second, a number of members of the elastase subfamily of serine hydrolases [27] were identified and clustered. Three chymotrypsin-like elastase family members 2A (CEL2A), 3A (CEL3A), 3B (CEL3B) and chymotrypsin (CTRC) formed a tight cluster indicating a similar expression pattern for the four proteins (Fig. 6). CEL3B showed a particularly high level in the ascending colon, and then declined gradually towards the distal regions (Fig. 7c). Conversely, leukocyte elastase inhibitor (ILEU), the endogenous elastase inhibitor [28], showed the lowest level in ascending and highest in rectum colon (Fig. 7d). A third protein cluster was the meprin family including two members meprin A subunit alpha (MEP1A) and meprin A subunit beta (MEP1B). The relative levels of both proteins were higher in proximal regions as compare to distal regions (Fig. 7e and 7f). In addition, there were two other proteins with low *P*-values as a biogeographic feature. Ectonucleotide pyrophosphatase/phosphodiesterase family member 7 (ENPP7) decreased from proximal to distal regions (Fig. 7g), yet carcinoembryonic antigen-related cell adhesion molecule 5 (CEAM5) increased expression in these same regions (Fig. 7h). In contrast to the foregoing, some known intestinal proteins demonstrated no significant biogeographic features, including mucin 2 (MUC2), trypsin-2 (TRY2), alpha-1-antitrypsin (A1AT), carboxypeptidase A1 (CBPA1), carboxypeptidase B (CBPB1), and intestinal-type alkaline phosphatase (PPBI) (Table S4).

## Discussion

To understand the basic biology and investigate host-microbial interaction at the human intestinal MLI, we used endoscopic lavage as a straightforward but novel methodology to sample the mucosal surface. This procedure involves minimal clinical risk, and yields analytically reproducible samples composed of both human and bacterial components expected at the MLI. Using high-throughput MALDI-TOF-MS proteomics and a suite of bioinformatics methods, we produced a high-dimensional dataset containing both human and bacteria peaks, which we termed the human mucosal metaproteome. Analysis of the mucosal metaproteome in healthy adults revealed substantial inter-individual variation, but also revealed a significant biogeographic feature. Furthermore, 49 distinct proteins were investigated in details in its biogeographic feature, and 4 protein networks were identified. These data suggest that MLI proteomics offer an informative, integrative strategy for studying the human mucosal ecosystem.

### A new way of viewing mucosal surface

Fecal or mucosal tissue biopsy samples are widely used in human mucosal studies, but have certain limitations. Whereas the fecal microbiota is a major biosynthetic and catabolic compartment with local and systemic physiologic effects [5], [6], it bears a microbial composition distinct from that of the mucosal surface [7], [8], [29], which instead is selected for adherence, host resistance, and mucosal trophic factors. Also, fecal samples are a mixture of products from all intestinal regions, which may obscure unique host-bacteria interactions present at individual sites along the mucosal interface [29]. Mucosal tissue biopsies are a useful biospecimen for mucosal luminal interaction, but since they require disruption of the mucosal barrier and are very small in surface area, they are suboptimal for proteomic and other functional analyses of the MLI. In comparison, mucosal lavage sampling is appealing because it spares disruption of the mucosal epithelium, permits repetitive sampling along the intestinal anatomy, and is abundant in biochemical yield for analysis. Recent studies have also shown that mucosal lavage yields robust recovery of surface microbiota whose composition associates with the host immunologic state and IBD disease state [30], [31].

Shotgun proteomics demonstrated that mucosal lavage recovered a diversity of both host and microbial proteins. With respect to the host, the predominance of identified MLI proteins were involved in binding and catalytic activity, comparable to a recent analysis of the mucosal transcriptome [23]. As might be anticipated, the MLI proteome was enriched compared to the transcriptome for extracellular proteins, and these were predominantly involved in response to stimulus and immune system processes. With regard to the abundant microbiome detected morphologically in the mucosal lavage specimens, it was surprising that identifiable peptides were predominantly of human proteins origin. However, shotgun proteomics preferably detects proteins with relatively high abundance, and hence may under-detect products from low frequency microbial taxa which in aggregate account for most of the microbial population [7], [8], [9]. Also, there is relatively incomplete documentation of bacterial proteins in the current protein database, particularly for poorly characterized taxa in the largely uncultured commensal microbiota. It is also notable that the shotgun analysis only identifies proteins of the microbes whose genomes have been sequenced and indexed in the database. Therefore, it is likely that the contribution from microbiota was underrepresented in our shotgun proteomic data.

Nevertheless, an interesting finding was the high number of identifications for bacterial proteins from two-component signaling systems. Two-component systems serve as a basic stimulus-response coupling mechanism to allow organisms to sense and respond to changes in many different environmental conditions [32]. A published bioinformatics study of phylogenetic distribution of nearly 5,000 histidine protein kinases from 207 sequenced prokaryotic genomes suggested that this two-component signaling system is crucial for the niche-adaption of bacteria [33]. Similarly, competition in the intestinal habitat for *Bacteroides thetaiotamicron* is exquisitely sensitive to the hybrid two-component system for efficient glycan utilization [14]. In this study, we not only identified proteins from *Bacteroidetes*, but also other bacterial phyla suggesting a more comprehensive role of the two-component system in intestinal microbiota.

### Integration of bioinformatics and proteomics

High-resolution, high-throughput MS proteomics presents a variety of challenges in data processing and analysis [16]. Without careful design and analysis, the results and conclusions from high-throughput proteomics can sometimes be misleading [18]. To overcome the lack of mature, commercially-available data management platforms, we established a unique data processing pipeline for the high-resolution MALDI-MS data from inception to final analysis. With regard to spectral data preprocessing procedures (binning, deisotoping, baseline subtraction, peak alignment, and intensity normalization), there are a variety of algorithms [34], [35], [36], [37], [38], [39], [40] and software [17], [41], [42] available. For this study, we selected a recent edition of SpecAlign [17] to process high-resolution spectrum data. In addition, we developed a stringent quality control protocol to remove outlier spectra. Consistent with other studies, we also found a batch effect in our MALDI-MS experiments [18]. To address this, we adopted an algorithm COMBAT [19], [43] written in R, which successfully corrected for the undesired batch effect before further analysis. We not only tested the reproducibility and robustness of our sampling strategy (sampling replicates), but also our working protocol (technical replicates). The results showed that our protocol includes all the important check-points for proteomic data management, and consistently produced high-quality spectral data for quantitative analysis. Since all the software and algorithms used in our protocol are open source and readily accessible to the public, we have tabulated the pipeline on our institutional website for the convenience of other investigators.

### Biogeographic features of mucosal metaproteome

A biogeographic feature of the microbiome [7] previously has been described in healthy subjects. However, a systemic study of the mucosa as an ecosystem at the colon surface has not yet been reported. Using mucosal lavage sampling, each subject was sampled 6 times at different colonic regions. The metaproteomic data revealed both inter-individual variation, and also a significant common biogeographic feature in the colon (distal versus proximal colonic regions). Interestingly, no age or gender related feature was observed. The distal colon is distinguished from more proximal colonic regions by the distinctive ultrastructure in the epithelial cells and the composition in the mucosal barrier [29], [44]. In addition, region-specific susceptibility is observed in ulcerative colitis and colon cancer. Thus, the biogeographic metaproteome may offer a functional counterpart to these distal colonic traits.

Quantitative shotgun proteomics established the identity and confirmed the biogeographic association of 49 host proteins demarcating the proximal and distal colon. These proteins clustered into several functional protein networks, including: immunoglobulin synthesis and transcytosis; the elastase subfamily of serine hydrolases and their inhibitors; and, members of the meprin metalloprotease family. Concordant with the latter observation, mucosal mRNA levels of meprin-α and meprin-β also decline from the proximal to distal colon. It is interesting to note that MEP1A l (meprin A) is a susceptibility gene for ulcerative colitis in human and immune colitis in mice [45]. In addition to these networks of proteins, strong biogeographic distributions were also observed for ENPP7 and CEAM5. ENPP7 is an ectoenzyme with sphingomyelin-specific phospholipase C specificity, that is released from the epithelial apical surface by luminal bile acid and trypsin-like activity [27]. As in our study, it also shows a decreasing expression gradient from ileum to distal colon [46], and has been associated with inflammation and tumorigenesis [47], [48], [49]. CEAM5 is a member of the carcinoembryonic antigen (CEA) family notable for epithelial cell adhesion and intracellular signaling, and colorectal carcinogenesis [50], [51], [52], [53]. CEAM5 mediates epithelial interaction with bacteria binding CEA-associated glycans, and its rapid physiologic exfoliation is considered a protective anti-microbial mechanism [54]. It is possible that its elevated abundance in the distal colon may reflect a role in interrupting bacterial intrusion in this region of exceptional luminal bacterial load.

In summary, this paper has described and validated an integrated proteomic approach that identifies functional features of the human mucosal surface. These include impressive distinctions between the proximal and distal colon, in accord with the concept that these regions are functional and developmentally distinct [55], [56]. This analytic approach offers a new and robust tool for understanding the basic biology and disease processes involving the MLI.

## Materials and Methods

### Mucosal lavage sample collection

All enrolled subjects were consented with a valid IRB protocol, and were prepared for colonoscopy by taking Golytely® the day before the procedure. During the colonoscopy procedure, 30 ml of sterile 0.9% saline was injected to the surface of each of the six different locations of the colon (cecum, ascending, transverse, descending, sigmoid, and rectum) (Fig. 1). The mucosal lavage samples were collected by vacuum suction with a Fujinon magnifying colonoscope. Typically, 20 ml of saline was recovered for each region. Lavage samples were kept on ice immediately after collection, and then transferred to the UCLA High-Throughput Clinical Proteomic Laboratory on the same day for processing.

### Cytology analysis

Mucosal lavage samples were spun at 1,000×g for 10 min to form a cell pellet. Pellets were fixed and sectioned following a standard cytology protocol by the UCLA Clinical Cytology Laboratory, and sections were stained by either H&E or Gram stain.

### Shotgun proteomic analysis

The supernatant from each mucosal lavage sample was spun at 4,000×g for 30 min to remove solid components, and the clear supernatant was transferred to a clean 50 ml tube, and acetone added to the supernatant to precipitate proteins and peptides. Samples were inverted several times to mix thoroughly and frozen at −80°C overnight. The following day, samples were thawed and spun down at 4,000×g for 30 min. The supernatant was discarded, and the precipitated pellet was dried at room temperature for 30 min. Each purified protein sample was dissolved in 1 ml 25 mM Tris-HCl buffer pH 8 with 2 M Urea by vortexing and pipetting. Samples were transferred to 2 ml protein LoBind microcentrifuge tubes (Eppendorf, Hamburg, Germany). The protein concentration of each combined sample was quantified using the Bradford reagent. 5 µl of 1 M dithiothreitol (DTT) was added to the remaining samples and incubated in 37°C bath for 1 hour. 50 µl aliquots of 0.5 M iodoacetamide (IAA) were added to the samples and incubated at room temperature with rocking for 1 hour in aluminum foil. After quenching with 10 µl of 1 M DTT and incubating at room temperature for 30 minutes, the samples were diluted two-fold to lower the DTT concentration. 10 µg of trypsin was added to each sample and incubated in a 37°C bath overnight. Post-digestion samples were run on a 4–18% Tris-Glycine gel to check the digestion efficacy. The digested samples were then cleaned-up through a 100 mg C18 cartridge (Alltech, Ontario, Canada), and then resuspended in 24 µl of 2% methylnitrile with 0.1% TFA solution. 10 µl of aliquots of each peptide sample was analyzed by liquid chromatography tandem mass spectrometer (LC-MS/MS) using an Eksigent autosampler coupled with Nano2DLC pump (Eksigent, Dublin, CA) and LTQ-Orbitrap (Thermo Fisher Scientific, Waltham, MA). The analytical column (10 cm×75 µm i.d.) contained 5 µm Integrafit Proteopep2 300 Å C18 (New Objective, Woburn, MA). Peptides were eluted using a HPLC gradient of 5% to 40% Buffer B in 45 min followed by a quick gradient of 40% to 90% Buffer B in 10 min, where Buffer A contains 0.1% formic acid in water and Buffer B contains 0.1% formic acid in acetonitrile. Mass spectra were collected in positive ion mode using the Orbitrap for parent mass determination and the LTQ for data dependent MS/MS acquisition of the top 5 most abundant peptides. MS/MS fragmentation spectra were searched using SEQUEST (Version v.27, rev. 12, Thermo Fisher Scientific) against the non-redundant Uniprot database indexed for human and bacteria proteins. Search parameters included carbamidomethyl cysteine (*C) as a static modification. Results derived from database searching were filtered using the following criteria: Xcorr >1.0(+1), 1.5(+2), 2(+3), and peptide probability score <0.001, and dCn >0.1 using Bioworks version 3.2 (Thermo Electron Corp).

### High-throughput MALDI-TOF-MS analysis and data processing

To each precipitated protein sample, 500 µl of PBS with 1% Triton-X was added and thoroughly mixed, and then transferred to a 2 ml microcentrifuge tubes. Samples were centrifuged at 10,000×g and the supernatant was collected. The concentration of each supernatant sample was determined by Bradford assay. 300 µg of total protein from each sample was diluted in PBS and applied to subsequent analyses. Samples were then passed through a 1-µm filter plate separately. 10 µl aliquots of the extracts were mixed with 200 µg of weak cation exchange (WCX) magnetic beads (MoBiTec, Goettingen, Germany) with 90 µl of 0.2 M ammonium acetate pH 4.0 with 0.01% TX-100. The process has been automated in a 96-well format with a Hamilton Starlet robot (Reno, NV) where the beads are pelleted on a strong plate magnet and washed 3 times. The beads were then desalted with 5 mM ammonium acetate and extracted with 15 µl of 1% trifluoroacetic acid. 10 µl of the extracts were removed and mixed with an equal volume of 5 mg/mL α-cyano-hydroxycinnamic acid matrix (CHCA) dissolved in 90% acetonitrile. 2 µl each of the extract-matrix mixture was then applied to a 96-well MALDI target in triplicates. After drying, the plate was read in a Perkin-Elmer Sciex prOTOF2000 reflectron mass spectrometer (San Jose, CA) with settings for optimal detection of peptides and small proteins between 2 and 20 kDa.

Since the samples were divided into 7 batches to complete the MALDI analyses, the potential batch effects was addressed by randomizing all samples, with each assigned an analysis date and the position on MALDI plate. In addition, 5 µl aliquots from each filtered sample were combined into a pooled sample, which was analyzed in quadruplicate on each plate to serve as a batch control of the inter- and intra-plate variation of MALDI analysis.

The pre-processing procedures are summarized in Fig. 3a. MALDI data were exported using PG600 prOTOF Loader software and cleaned in Python software using a homemade script. This initial data cleaning step was critical to render the MALDI-TOF-MS data accessible for the subsequent visualization and pre-analytic processing in SpecAlign software (http://physchem.ox.ac.uk/~jwong/specalign/index.htm). Each spectrum was first trimmed to 2,000 to 20,000 m/z range, and binned at 0.25 m/z window size to reduce the data dimension to a manageable size. All spectra were then loaded simultaneously into SpecAlign. Combining of all isotopic peaks was achieved by smoothing the spectrum at 5 m/z window size twice. Baseline subtraction was performed using 5 m/z window size. Stringent quality control (QC) rules were applied to pick out spectra with poor qualities. Each spectrum was inspected individually at two levels: total ion intensity level and average correlation with the entire dataset. Spectra with both intensity level and average correlation in the bottom 20% of the spectral profiles were disqualified. The remaining spectra were then normalized to total ion current (TIC). Peaks were identified using criteria of signal threshold 0.5, window size of 5 m/z, and signal-to-noise ratio of 1.5. Average spectra of all technical replicates from the same sample were produced in Python using a homemade script. All scripts are downloadable from our laboratory website.

### Quantitative shotgun analysis and data processing

Fifteen supernatant samples previously analyzed by MALDI-MS were selected for quantitative shotgun analysis. It is the same as described earlier with the exception of performing in-gel trypsin digestion instead of in-solution. Briefly, 50 ug proteins of each sample were loaded onto a NuPAGE 4–12% Bis-tris gel. The gel was run at 100 V for about 15 minutes to allow the proteins to migrate through the stacking portion of the gel. The entire lane was excised, reduced with DTT, alkylated with IAA, and digested with trypsin at a 1∶50 ratio. We did notice that the in-gel digestion greatly increased the efficacy of trypsin, and it was probably due to inactivation of the trypsin inhibitor A1AT present at large amount in the lavage samples.

All MS/MS spectra were then analyzed using Mascot (Version 2.2.2, Matrix Science, London, UK), and searched against the SwissProt 57.15 database (selected for Homo sapiens only, 20266 entries). Mascot was searched with a fragment ion mass tolerance of 0.40 Da and a parent ion tolerance of 10.0 PPM. Iodoacetamide derivative of cysteine was specified in Mascot as a fixed modification. Oxidation of methionine was specified in Mascot as a variable modification. Scaffold (Proteome Software Inc., Portland, OR) was used to validate MS/MS based peptide and protein identifications. Peptide identifications were accepted if they exceeded specific database search engine thresholds. Mascot identifications required at least ion scores must be greater than both the associated identity scores and 20, 30, 40 and 40 for singly, doubly, triply and quadruply charged peptides. Protein identifications were accepted if they contained at least 1 identified peptide. Proteins that contained similar peptides and could not be differentiated based on MS/MS analysis alone were grouped to satisfy the principles of parsimony.

The following quality control criteria were further implemented to select candidate proteins: 1) Minimal peptide hits had to be 2 for each protein. 2) Sample U053 and U188 were removed as the total spectrum counts were significantly lower than the others. 3) Each protein had to be present in more than 50% of the samples. 49 proteins were filtered out for subsequent statistical analysis. The label-free spectrum counting method was adopted to quantify the relative abundance of protein in analyzing shotgun proteomic data [22], [25], [26]. Normalization factor was calculated for each sample by dividing the total spectrum counts by the average spectrum counts. The spectrum count of a specific protein was multiplied by the normalization factor to calculate the relative abundance. The list of the normalized abundance and the *P*-values from ANOVA for biogeographic effect of the 49 selected proteins is shown in Table S4.

### Statistics

All statistical analyses were conducted in R software (http://www.r-project.org/). The averaged spectrum of each sample was loaded into R. For the MALDI-MS data, Pearson correlation coefficient was used to evaluate the batch effect, which was later corrected using the COMBAT package [19], [43]. Principal Variance Component Analysis (PVCA) was used to calculate the inter- and intra-subject variability in the metaproteome, and the significance of the difference was further evaluated by permutation test and NLME analysis. For permutation test, an average spectrum for each region was calculated by averaging the intensity of each peak within the same group, e.g. anatomic region, and gender. The differences between two groups were measured by the correlation coefficient between averaged spectra. The spectral data was then permutated 1000 times, and the difference between two groups was calculated after each permutation. The number of distance smaller than the observed distance was divided by 1000 to give the *P*-value of the specific group feature in examination. For NLME analysis, the individual factor was set as the random effect in the model, and the region, gender, and age were set as the fixed effects. For the quantitative shotgun data, Z-score transformation was carried out within each protein, and the normalized levels of the 49 proteins identified were visualized in a heatmap with hierarchical clustering. The *P*-value of the biogeographic feature for each protein was calculated by ANOVA. All scripts written in R are available from our laboratory website.

## Supporting Information

Table S1 Proteins identified with shotgun proteomic analysis.
(RTF)

Table S2 Correlation matrix of the 28 quality control samples from 7 batches.
(RTF)

Table S3 *P*-values of all protein/peptide features in the NLME analysis.
(RTF)

Table S4 *P*-values of 49 proteins with biogeographic feature.
(RTF)

Dataset S1 Metaproteome MALDI dataset containing relative ion intensities of 438 peaks from 203 samples.
(CSV)

Dataset S2 Metaprotome shotgun dataset containing spectrum counts of 300 proteins from 20 samples.
(CSV)
