# Supplementary material for: A Metaproteomic Approach to Study Human-Microbial Ecosystems at the Mucosal Luminal Interface
Source: PLoS One. 2011 Nov 21;6(11):e26542. doi: 10.1371/journal.pone.0026542 (PMC3221670; doi:10.1371/journal.pone.0026542)
Supplement: Table S1 — Proteins identified with shotgun proteomic analysis. (RTF) [file pone.0026542.s001.rtf]

Table S1. Proteins identified in the shotgun proteomic analysis
Run#	Reference Name	Origin	Phylogenetic origin	
A	gi|94967405|ref|YP_589453.1| two component, sigma54 specific, Fis family transcriptional regulator 	Candidatus Koribacter versatilis Ellin345	Bacteria; Acidobacteria; Candidatus Koribacter.	
B	gi|94969349|ref|YP_591397.1| radical SAM family Fe-S protein 	Acidobacteria bacterium Ellin345	Bacteria; Acidobacteria; Candidatus Koribacter.	
B	gi|94969498|ref|YP_591546.1| butyryl-CoA dehydrogenase 	Acidobacteria bacterium Ellin345	Bacteria; Acidobacteria; Candidatus Koribacter.	
A	gi|94970740|ref|YP_592788.1| two component LuxR family transcriptional regulator 	Candidatus Koribacter versatilis Ellin345	Bacteria; Acidobacteria; Candidatus Koribacter.	
B	gi|126661749|ref|ZP_01732748.1| preprotein translocase, secA subunit 	Flavobacteria bacterium BAL38	Bacteria; Bacteroidetes; Flavobacteria; Flavobacteriales.	
A	gi|126662825|ref|ZP_01733824.1| hypothetical protein FBBAL38_05700 	Flavobacteria bacterium BAL38	Bacteria; Bacteroidetes; Flavobacteria; Flavobacteriales.	
B	gi|126664137|ref|ZP_01735130.1| aminopeptidase N 	Flavobacteria bacterium BAL38	Bacteria; Bacteroidetes; Flavobacteria; Flavobacteriales.	
A	gi|163785941|ref|ZP_02180389.1| putative two-component system sensor, no kinase domain 	Flavobacteria bacterium ALC-1	Bacteria; Bacteroidetes; Flavobacteria; Flavobacteriales.	
A	gi|163786016|ref|ZP_02180464.1| two-component system sensor histidine kinase/response 	Flavobacteria bacterium ALC-1	Bacteria; Bacteroidetes; Flavobacteria; Flavobacteriales.	
B	gi|163786538|ref|ZP_02180986.1| hypothetical protein FBALC1_15172 	Flavobacteriales bacterium ALC-1	Bacteria; Bacteroidetes; Flavobacteria; Flavobacteriales.	
A	gi|163787935|ref|ZP_02182381.1| hypothetical protein FBALC1_06138 	Flavobacteriales bacterium ALC-1	Bacteria; Bacteroidetes; Flavobacteria; Flavobacteriales.	
B	gi|225010134|ref|ZP_03700606.1| tRNA (guanine-N(7)-)-methyltransferase 	Flavobacteria bacterium MS024-3C	Bacteria; Bacteroidetes; Flavobacteria; Flavobacteriales.	
B	gi|225011154|ref|ZP_03701616.1| putative molybdopterin oxidoreductase, iron-sulfur binding subunit 	Flavobacteria bacterium MS024-3C	Bacteria; Bacteroidetes; Flavobacteria; Flavobacteriales.	
A	gi|225011537|ref|ZP_03701975.1| hypothetical protein Flav2ADRAFT_1320 	Flavobacteria bacterium MS024-2A	Bacteria; Bacteroidetes; Flavobacteria; Flavobacteriales.	
B	gi|225012793|ref|ZP_03703227.1| peptidase, family M14 	Flavobacteria bacterium MS024-2A	Bacteria; Bacteroidetes; Flavobacteria; Flavobacteriales.	
B	gi|88711061|ref|ZP_01105149.1| molybdopterin oxidoreductase, iron-sulfur binding subunit 	Flavobacteriales bacterium HTCC2170	Bacteria; Bacteroidetes; Flavobacteria; Flavobacteriales.	
A	gi|88712002|ref|ZP_01106089.1| acyl-CoA dehydrogenase 	Flavobacteriales bacterium HTCC2170	Bacteria; Bacteroidetes; Flavobacteria; Flavobacteriales.	
B	gi|88713661|ref|ZP_01107743.1| putative dephospho-CoA kinase 	Flavobacteriales bacterium HTCC2170	Bacteria; Bacteroidetes; Flavobacteria; Flavobacteriales.	
B	gi|88713714|ref|ZP_01107795.1| deoxyribose-phosphate aldolase 	Flavobacteriales bacterium HTCC2170	Bacteria; Bacteroidetes; Flavobacteria; Flavobacteriales.	
A	gi|89890002|ref|ZP_01201513.1| DNA /RNA helicase, superfamily I 	Flavobacteria bacterium BBFL7	Bacteria; Bacteroidetes; Flavobacteria; Flavobacteriales.	
A	gi|89890227|ref|ZP_01201737.1| conserved hypothetical protein 	Flavobacteria bacterium BBFL7	Bacteria; Bacteroidetes; Flavobacteria; Flavobacteriales.	
B	gi|145220364|ref|YP_001131073.1| translation initiation factor IF-2  	Prosthecochloris vibrioformis DSM 265	Bacteria; Chlorobi; Chlorobia; Chlorobiales; Chlorobiaceae;  Chlorobium/Pelodictyon group; Chlorobium.	
B	gi|224401691|ref|YP_002617358.1| RNA polymerase sigma factor, cyanobacterial RpoD-like family 	Microcoleus chthonoplastes PCC 7420	Bacteria; Cyanobacteria; Oscillatoriales; Microcoleus.Bacteria; Cyanobacteria; Oscillatoriales; Microcoleus.	
A	gi|88713174|ref|ZP_01107258.1| UDP-N-acetylmuramoylalanyl-D-glutamate--2,6-diaminopimelate ligase 	Bacillus cereus E33L	Bacteria; Firmicutes; Bacillales; Bacillaceae; Bacillus; Bacillus; cereus group.	
A	gi|30018667|ref|NP_830298.1| transporter family protein 	Bacillus cereus ATCC 14579	Bacteria; Firmicutes; Bacillales; Bacillaceae; Bacillus; Bacillus; cereus group.	
B	gi|69932979|ref|ZP_00628181.1| Ribosomal protein S19, bacterial and organelle form 	Paracoccus denitrificans PD1222	Bacteria; Proteobacteria; Alphaproteobacteria; Rhodobacterales; Rhodobacteraceae; Paracoccus.	
A	gi|167850938|ref|ZP_02476446.1| bacterial extracellular solute-binding protein, family 3 	Burkholderia pseudomallei B7210	Bacteria; Proteobacteria; Betaproteobacteria; Burkholderiales; Burkholderiaceae; Burkholderia; pseudomallei group.	
B	gi|224367358|ref|YP_002601521.1| putative glycogen synthase (starch bacterial glycogen synthase) 	Desulfobacterium autotrophicum HRM2	Bacteria; Proteobacteria; Deltaproteobacteria; Desulfobacterales; Desulfobacteraceae; Desulfobacterium.	
A	gi|18655553|pdb|1KN9|A Chain A, Crystal Structure Of A Bacterial Signal Peptidase Apo-Enzyme 	Escherichia coli K-12	Bacteria; Proteobacteria; Gammaproteobacteria; Enterobacteriales; Enterobacteriaceae; Escherichia.	
A	gi|9955210|pdb|1DVO|A Chain A, The X-Ray Crystal Structure Of Fino, A Repressor Of Bacterial Conjugation 	Escherichia coli	Bacteria; Proteobacteria; Gammaproteobacteria; Enterobacteriales; Enterobacteriaceae; Escherichia.	
B	gi|13449190|ref|NP_085406.1| virulence protein 	Shigella flexneri	Bacteria; Proteobacteria; Gammaproteobacteria; Enterobacteriales; Enterobacteriaceae; Shigella.	
B	gi|52842028|ref|YP_095827.1| RecA DNA recombination protein  	Legionella pneumophila subsp. pneumophila str. Philadelphia 1	Bacteria; Proteobacteria; Gammaproteobacteria; Legionellales; Legionellaceae; Legionella.	
B	gi|223479148|ref|YP_002583938.1| Bacterial lipid A biosynthesis acyltransferase superfamily 	Alcanivorax sp. DG881	Bacteria; Proteobacteria; Gammaproteobacteria; Oceanospirillales; Alcanivoracaceae; Alcanivorax.	
A	gi|83647078|ref|YP_435513.1| hypothetical protein HCH_04383 	Hahella chejuensis KCTC 2396	Bacteria; Proteobacteria; Gammaproteobacteria; Oceanospirillales; Hahellaceae; Hahella.	
B	gi|71275910|ref|ZP_00652193.1| RecA bacterial DNA recombination protein 	Xylella fastidiosa Dixon	Bacteria; Proteobacteria; Gammaproteobacteria; Xanthomonadales; Xanthomonadaceae; Xylella.	
B	gi|28829090|gb|AAO51654.1| similar to Homo sapiens (Human). KIAA0041 protein (Fragment) 	Dictyostelium discoideum	Eukaryota; Amoebozoa; Mycetozoa; Dictyosteliida; Dictyostelium.	
B	gi|66818515|ref|XP_642917.1| RhoGAP domain-containing protein 	Dictyostelium discoideum AX4	Eukaryota; Amoebozoa; Mycetozoa; Dictyosteliida; Dictyostelium.	
A	gi|66823209|ref|XP_644959.1| WD40 repeat-containing protein 	Dictyostelium discoideum AX4	Eukaryota; Amoebozoa; Mycetozoa; Dictyosteliida; Dictyostelium.	
B	gi|66824379|ref|XP_645544.1| peptidase M16 family protein 	Dictyostelium discoideum AX4	Eukaryota; Amoebozoa; Mycetozoa; Dictyosteliida; Dictyostelium.	
B	gi|52627189|ref|NP_001005318.1| hypothetical protein LOC368995 	Danio rerio	Eukaryota; Metazoa; Chordata; Craniata; Vertebrata; Euteleostomi; Actinopterygii; Neopterygii; Teleostei; Ostariophysi; Cypriniformes; Cyprinidae; Danio.	
B	gi|124359286|gb|ABD28418.2| Ribosomal protein L20, bacterial and organelle form 	Medicago truncatula	Eukaryota; Viridiplantae; Streptophyta; Embryophyta; Tracheophyta;Spermatophyta; Magnoliophyta; eudicotyledons; core eudicotyledons;rosids; fabids; Fabales; Fabaceae; Papilionoideae; Trifolieae; Medicago	
B	gi|18249906|ref|NP_543094.1| hypothetical protein P27p42 	Enterobacteria phage phiP27	Viruses; dsDNA viruses, no RNA stage; Caudovirales; Myoviridae.	
A	gi|45686334|ref|YP_003919.1| putative tail fiber 	Enterobacteria phage T1	Viruses; dsDNA viruses, no RNA stage; Caudovirales; Myoviridae.	
B	gi|9630336|ref|NP_046765.1| gpK 	Enterobacteria phage P2	Viruses; dsDNA viruses, no RNA stage; Caudovirales; Myoviridae; P2-like viruses.	
A	gi|10437384|dbj|BAB15043.1| unnamed protein product 	Homo sapiens	
A	gi|10645195|ref|NP_066390.1| histone cluster 1, H2ae 	Homo sapiens	
A	gi|1082509|pir||S46391 Ig heavy chain V region 	Homo sapiens	
B	gi|119368828|sp|Q96MU6.2|ZN778_HUMAN RecName: Full=Zinc finger protein 778 	Homo sapiens	
B	gi|119372315|ref|NP_001073278.1| xin actin-binding repeat containing 2 isoform 2 	Homo sapiens	
A	gi|119590790|gb|EAW70384.1| hCG2012684 	Homo sapiens	
A	gi|119591205|gb|EAW70799.1| hCG2012440 	Homo sapiens	
A	gi|119606974|gb|EAW86568.1| hCG22737 	Homo sapiens	
B	gi|119608095|gb|EAW87689.1| tetratricopeptide repeat domain 16, isoform CRA_c 	Homo sapiens	
B	gi|119609651|gb|EAW89245.1| hematological and neurological expressed 1, isoform CRA_b 	Homo sapiens	
B	gi|119621904|gb|EAX01499.1| zinc finger protein 519, isoform CRA_c 	Homo sapiens	
A	gi|119623011|gb|EAX02606.1| hCG1994947, isoform CRA_c 	Homo sapiens	
B	gi|119628459|gb|EAX08054.1| catenin (cadherin-associated protein), delta 2 (neural plakophilin-related arm-repeat protein), isoform CRA_b 	Homo sapiens	
B	gi|13124879|ref|NP_002465.1| smooth muscle myosin heavy chain 11 isoform SM1A 	Homo sapiens	
A	gi|13171332|gb|AAK13628.1|AF234252_1 immunoglobulin heavy chain variable region 	Homo sapiens	
A	gi|13376842|ref|NP_079511.1| tankyrase, TRF1-interacting ankyrin-related ADP-ribose polymerase 2 	Homo sapiens	
A	gi|14042668|dbj|BAB55345.1| unnamed protein product 	Homo sapiens	
A	gi|14149738|ref|NP_065777.1| neurolysin 	Homo sapiens	
A	gi|14195655|sp|P08861.2|ELA3B_HUMAN RecName: Full=Elastase-3B 	Homo sapiens	
A	gi|157266292|ref|NP_001622.2| intestinal alkaline phosphatase precursor 	Homo sapiens	
A	gi|157388923|ref|NP_060417.2| molybdenum cofactor sulfurase 	Homo sapiens	
B	gi|15826862|ref|NP_296375.1| coiled-coil domain containing 120 	Homo sapiens	
A	gi|166235167|ref|NP_055230.2| zinc finger protein ZNF-U69274 	Homo sapiens	
A	gi|169209605|ref|XP_001721015.1| PREDICTED: hypothetical protein 	Homo sapiens	
B	gi|169214746|ref|XP_001718002.1| PREDICTED: hypothetical protein 	Homo sapiens	
B	gi|17066105|emb|CAD12456.1| Titin 	Homo sapiens	
B	gi|17318559|ref|NP_001229.1| cyclin E1 isoform 1 	Homo sapiens	
A	gi|17648144|gb|AAC39568.2| maltase-glucoamylase 	Homo sapiens	
A	gi|179531|gb|AAA35607.1| IgE-binding protein 	Homo sapiens	
A	gi|189054206|dbj|BAG36726.1| unnamed protein product 	Homo sapiens	
A	gi|194375377|dbj|BAG62801.1| unnamed protein product 	Homo sapiens	
A	gi|194380508|dbj|BAG58407.1| unnamed protein product 	Homo sapiens	
B	gi|197115096|emb|CAR62552.1| immunoglobulin heavy chain variable region 	Homo sapiens	
A	gi|21361454|ref|NP_037460.2| pyrroline-5-carboxylate reductase family, member 2 	Homo sapiens	
B	gi|21928822|dbj|BAC05997.1| seven transmembrane helix receptor 	Homo sapiens	
A	gi|24474791|emb|CAD43720.1| small nuclear ribonucleoprotein component 	Homo sapiens	
B	gi|2833252|sp|Q14571.1|ITPR2_HUMAN RecName: Full=Inositol 1,4,5-trisphosphate receptor type 2 	Homo sapiens	
B	gi|29653|emb|CAA45535.1| putative oncogene 	Homo sapiens	
A	gi|29791717|gb|AAH50597.1| PDCD6 protein 	Homo sapiens	
B	gi|32698918|ref|NP_872349.1| NOL1/NOP2/Sun domain family, member 6 	Homo sapiens	
A	gi|33187651|gb|AAP97680.1|AF450487_1 kinesin-like protein KIF2 	Homo sapiens	
B	gi|34528869|dbj|BAC85589.1| unnamed protein product 	Homo sapiens	
B	gi|34531580|dbj|BAC86173.1| unnamed protein product 	Homo sapiens	
B	gi|38348344|ref|NP_940917.1| hypothetical protein LOC374355 	Homo sapiens	
A	gi|3892640|dbj|BAA34537.1| glucuronyltransferase I 	Homo sapiens	
A	gi|4009458|gb|AAC95428.1| calcium-dependent chloride channel-1 	Homo sapiens	
B	gi|40788231|dbj|BAA20828.2| KIAA0373 	Homo sapiens	
B	gi|4261903|gb|AAD14203.1|S75992_1 voltage-dependent sodium channel alpha subunit 	Homo sapiens	
A	gi|4501885|ref|NP_001092.1| beta actin 	Homo sapiens	
B	gi|4502923|ref|NP_001830.1| calponin 3 	Homo sapiens	
A	gi|4504255|ref|NP_002097.1| H2A histone family, member Z 	Homo sapiens	
B	gi|4504301|ref|NP_003529.1| histone cluster 1, H4a 	Homo sapiens	
A	gi|4504447|ref|NP_002128.1| heterogeneous nuclear ribonucleoprotein A2/B1 isoform A2 	Homo sapiens	
B	gi|4504993|ref|NP_002301.1| leukemia inhibitory factor receptor precursor 	Homo sapiens	
A	gi|4505683|ref|NP_002600.1| platelet-derived growth factor receptor beta precursor 	Homo sapiens	
B	gi|4505719|ref|NP_003837.1| peroxisomal biogenesis factor 11 beta 	Homo sapiens	
A	gi|4507687|ref|NP_003296.1| transient receptor potential cation channel, subfamily C, member 3 isoform B 	Homo sapiens	
A	gi|4758874|ref|NP_004791.1| transmembrane 9 superfamily member 2 	Homo sapiens	
A	gi|50949260|emb|CAH10352.1| Ka35 protein 	Homo sapiens	
B	gi|51094682|gb|EAL23932.1| hypothetical protein FLJ10300 	Homo sapiens	
B	gi|51094903|gb|EAL24148.1| hypothetical protein DKFZp564O0523 	Homo sapiens	
A	gi|56208513|emb|CAI20086.1| chromosome 22 open reading frame 34 	Homo sapiens	
B	gi|58082081|ref|NP_001010855.1| phosphoinositide-3-kinase, regulatory subunit 6 	Homo sapiens	
B	gi|7020908|dbj|BAA91316.1| unnamed protein product 	Homo sapiens	
B	gi|7243115|dbj|BAA92605.1| KIAA1367 protein 	Homo sapiens	
B	gi|74717598|sp|Q9BXB7.2|SPT16_HUMAN RecName: Full=Spermatogenesis-associated protein 16 	Homo sapiens	
B	gi|74761835|sp|Q9NZI8.1|IF2B1_HUMAN RecName: Full=Insulin-like growth factor 2 mRNA-binding protein 	Homo sapiens	
A	gi|7669492|ref|NP_002037.2| glyceraldehyde-3-phosphate dehydrogenase 	Homo sapiens	
B	gi|76800646|sp|Q9Y4K1.3|AIM1_HUMAN RecName: Full=Absent in melanoma 1 protein 	Homo sapiens	
A	gi|7706693|ref|NP_056968.1| membrane-bound transcription factor peptidase, site 2 	Homo sapiens	
B	gi|8099346|gb|AAF72103.1|AF154845_1 MARK 	Homo sapiens	
A	gi|88698088|gb|ABD48898.1| mitochondrial translational release factor 1-like 	Homo sapiens	
B	gi|89886447|ref|NP_001034813.1| nitric oxide synthase trafficker isoform 2 	Homo sapiens	
A	gi|9910476|ref|NP_064553.1| p21-activated kinase 6 	Homo sapiens	
