# Supplementary material for: A Metaproteomic Approach to Study Human-Microbial Ecosystems at the Mucosal Luminal Interface
Source: PLoS One. 2011 Nov 21;6(11):e26542. doi: 10.1371/journal.pone.0026542 (PMC3221670; doi:10.1371/journal.pone.0026542)
Supplement: Table S2 — Correlation matrix of the 28 quality control samples from 7 batches. (RTF) [file pone.0026542.s002.rtf]

	A1	A2	A3	A4	B1	B2	B3	B4	C1	C2	C3	C4	D1	D2	D3	D4	E1	E2	E3	E4	F1	F2	F3	F4	G1	G2	G3	G4	
A1	1.00	0.97	0.96	0.99	0.97	0.97	0.95	0.95	0.94	0.94	0.94	0.94	0.98	0.98	0.96	0.98	0.94	0.94	0.94	0.94	0.99	0.97	0.97	0.93	0.93	0.94	0.94	0.94	
A2	0.97	1.00	1.00	0.98	0.99	0.99	0.97	0.98	0.98	0.98	0.98	0.98	0.98	0.92	0.88	0.98	0.98	0.98	0.98	0.98	0.97	0.98	0.98	0.96	0.97	0.98	0.98	0.98	
A3	0.96	1.00	1.00	0.98	0.98	0.98	0.95	0.97	0.98	0.99	0.99	0.99	0.98	0.90	0.85	0.97	0.99	0.99	0.98	0.98	0.96	0.97	0.97	0.94	0.97	0.99	0.99	0.98	
A4	0.99	0.98	0.98	1.00	0.98	0.98	0.95	0.96	0.96	0.97	0.97	0.97	0.99	0.97	0.94	0.98	0.97	0.97	0.97	0.97	0.99	0.98	0.98	0.94	0.96	0.97	0.97	0.96	
B1	0.97	0.99	0.98	0.98	1.00	1.00	0.98	0.99	0.97	0.97	0.98	0.98	0.99	0.94	0.91	1.00	0.98	0.98	0.98	0.98	0.99	0.99	1.00	0.97	0.97	0.98	0.98	0.98	
B2	0.97	0.99	0.98	0.98	1.00	1.00	0.98	0.99	0.97	0.97	0.98	0.98	0.99	0.93	0.90	0.99	0.98	0.98	0.98	0.98	0.99	0.99	1.00	0.97	0.98	0.98	0.98	0.98	
B3	0.95	0.97	0.95	0.95	0.98	0.98	1.00	1.00	0.93	0.93	0.93	0.93	0.96	0.90	0.89	0.99	0.93	0.94	0.94	0.93	0.97	0.98	0.99	0.98	0.93	0.94	0.94	0.93	
B4	0.95	0.98	0.97	0.96	0.99	0.99	1.00	1.00	0.95	0.95	0.96	0.96	0.97	0.91	0.88	0.99	0.96	0.96	0.96	0.96	0.98	0.99	0.99	0.98	0.95	0.96	0.96	0.96	
C1	0.94	0.98	0.98	0.96	0.97	0.97	0.93	0.95	1.00	0.99	1.00	1.00	0.98	0.90	0.85	0.96	0.99	0.99	0.99	1.00	0.95	0.96	0.97	0.93	0.99	1.00	1.00	0.99	
C2	0.94	0.98	0.99	0.97	0.97	0.97	0.93	0.95	0.99	1.00	1.00	0.99	0.98	0.90	0.85	0.96	0.99	0.99	0.99	0.99	0.95	0.96	0.96	0.92	0.98	0.99	0.99	0.98	
C3	0.94	0.98	0.99	0.97	0.98	0.98	0.93	0.96	1.00	1.00	1.00	1.00	0.98	0.91	0.85	0.96	1.00	1.00	1.00	0.99	0.95	0.96	0.97	0.93	0.99	1.00	1.00	0.99	
C4	0.94	0.98	0.99	0.97	0.98	0.98	0.93	0.96	1.00	0.99	1.00	1.00	0.98	0.91	0.85	0.96	1.00	1.00	1.00	1.00	0.95	0.97	0.97	0.93	0.99	1.00	1.00	1.00	
D1	0.98	0.98	0.98	0.99	0.99	0.99	0.96	0.97	0.98	0.98	0.98	0.98	1.00	0.97	0.93	0.99	0.98	0.98	0.98	0.98	0.99	0.99	0.99	0.95	0.97	0.98	0.98	0.98	
D2	0.98	0.92	0.90	0.97	0.94	0.93	0.90	0.91	0.90	0.90	0.91	0.91	0.97	1.00	0.99	0.95	0.91	0.91	0.91	0.90	0.97	0.95	0.94	0.89	0.90	0.91	0.91	0.90	
D3	0.96	0.88	0.85	0.94	0.91	0.90	0.89	0.88	0.85	0.85	0.85	0.85	0.93	0.99	1.00	0.93	0.86	0.86	0.85	0.85	0.96	0.93	0.91	0.87	0.85	0.85	0.85	0.85	
D4	0.98	0.98	0.97	0.98	1.00	0.99	0.99	0.99	0.96	0.96	0.96	0.96	0.99	0.95	0.93	1.00	0.97	0.97	0.97	0.97	1.00	1.00	1.00	0.97	0.96	0.97	0.97	0.97	
E1	0.94	0.98	0.99	0.97	0.98	0.98	0.93	0.96	0.99	0.99	1.00	1.00	0.98	0.91	0.86	0.97	1.00	1.00	1.00	1.00	0.96	0.97	0.97	0.93	0.99	1.00	1.00	1.00	
E2	0.94	0.98	0.99	0.97	0.98	0.98	0.94	0.96	0.99	0.99	1.00	1.00	0.98	0.91	0.86	0.97	1.00	1.00	1.00	1.00	0.96	0.97	0.97	0.93	0.99	1.00	1.00	1.00	
E3	0.94	0.98	0.98	0.97	0.98	0.98	0.94	0.96	0.99	0.99	1.00	1.00	0.98	0.91	0.85	0.97	1.00	1.00	1.00	1.00	0.96	0.97	0.97	0.93	1.00	1.00	1.00	1.00	
E4	0.94	0.98	0.98	0.97	0.98	0.98	0.93	0.96	1.00	0.99	0.99	1.00	0.98	0.90	0.85	0.97	1.00	1.00	1.00	1.00	0.95	0.97	0.97	0.93	1.00	1.00	1.00	1.00	
F1	0.99	0.97	0.96	0.99	0.99	0.99	0.97	0.98	0.95	0.95	0.95	0.95	0.99	0.97	0.96	1.00	0.96	0.96	0.96	0.95	1.00	0.99	0.99	0.96	0.95	0.95	0.95	0.95	
F2	0.97	0.98	0.97	0.98	0.99	0.99	0.98	0.99	0.96	0.96	0.96	0.97	0.99	0.95	0.93	1.00	0.97	0.97	0.97	0.97	0.99	1.00	1.00	0.98	0.96	0.97	0.97	0.96	
F3	0.97	0.98	0.97	0.98	1.00	1.00	0.99	0.99	0.97	0.96	0.97	0.97	0.99	0.94	0.91	1.00	0.97	0.97	0.97	0.97	0.99	1.00	1.00	0.98	0.97	0.97	0.97	0.97	
F4	0.93	0.96	0.94	0.94	0.97	0.97	0.98	0.98	0.93	0.92	0.93	0.93	0.95	0.89	0.87	0.97	0.93	0.93	0.93	0.93	0.96	0.98	0.98	1.00	0.93	0.93	0.93	0.93	
G1	0.93	0.97	0.97	0.96	0.97	0.98	0.93	0.95	0.99	0.98	0.99	0.99	0.97	0.90	0.85	0.96	0.99	0.99	1.00	1.00	0.95	0.96	0.97	0.93	1.00	0.99	1.00	1.00	
G2	0.94	0.98	0.99	0.97	0.98	0.98	0.94	0.96	1.00	0.99	1.00	1.00	0.98	0.91	0.85	0.97	1.00	1.00	1.00	1.00	0.95	0.97	0.97	0.93	0.99	1.00	1.00	1.00	
G3	0.94	0.98	0.99	0.97	0.98	0.98	0.94	0.96	1.00	0.99	1.00	1.00	0.98	0.91	0.85	0.97	1.00	1.00	1.00	1.00	0.95	0.97	0.97	0.93	1.00	1.00	1.00	1.00	
G4	0.94	0.98	0.98	0.96	0.98	0.98	0.93	0.96	0.99	0.98	0.99	1.00	0.98	0.90	0.85	0.97	1.00	1.00	1.00	1.00	0.95	0.96	0.97	0.93	1.00	1.00	1.00	1.00	
Table S2. Correlation matrix of the 28 quality control samples from 7 batches
