# Supplementary material for: A Metaproteomic Approach to Study Human-Microbial Ecosystems at the Mucosal Luminal Interface
Source: PLoS One. 2011 Nov 21;6(11):e26542. doi: 10.1371/journal.pone.0026542 (PMC3221670; doi:10.1371/journal.pone.0026542)
Supplement: Table S3 — P-values of all protein/peptide features in the NLME analysis. (RTF) [file pone.0026542.s003.rtf]

Table S3. P-values of all protein/peptide features in the NLME analysis
Protein/peptide peaks (m/z)	P-value for biogeographic effect	P-value for gender effect	P-value for age effect	
2017.21	1.31E-02	0.683142	0.541197	
2024.57	2.16E-02	0.939921	0.059148	
2032.97	2.79E-03	0.587424	0.639793	
2037.04	1.20E-02	0.555251	0.461097	
2044.69	2.40E-01	0.635485	0.741815	
2052.1	2.28E-01	0.71561	0.538498	
2061.06	5.67E-01	0.972013	0.058853	
2068.25	1.54E-01	0.157266	0.468763	
2076.21	2.06E-01	0.153939	0.481969	
2084.2	1.29E-01	0.745238	0.931578	
2099.43	2.75E-02	0.338158	0.962264	
2104.09	5.96E-05	0.739361	0.192629	
2118.1	5.56E-01	0.550892	0.307132	
2130.33	4.90E-02	0.139306	0.558915	
2141.55	6.17E-03	0.25642	0.284289	
2147.04	8.46E-01	0.749314	0.26965	
2153.58	6.70E-02	0.834965	0.980461	
2159.09	2.70E-01	0.115284	0.27067	
2166.44	8.15E-02	0.491789	0.792781	
2177.76	9.99E-03	0.979623	0.510534	
2185.4	1.41E-03	0.572314	0.918238	
2198.36	1.65E-02	0.20577	0.75317	
2218.26	9.77E-02	0.593596	0.435151	
2233.17	1.79E-02	0.151303	0.437977	
2245.46	3.55E-02	0.840402	0.743748	
2246.26	4.05E-01	0.512561	0.327931	
2262.33	7.39E-01	0.677045	0.047953	
2269.86	1.57E-03	0.611744	0.727927	
2284.94	5.43E-02	0.745863	0.966933	
2289.23	9.46E-02	0.287969	0.653731	
2289.73	9.97E-02	0.43217	0.83752	
2299.32	3.16E-03	0.568124	0.702389	
2314.26	7.44E-01	0.767888	0.70869	
2332.29	8.33E-02	0.361415	0.326725	
2345.29	1.19E-02	0.519	0.375735	
2357.3	5.66E-02	0.49628	0.663955	
2360.37	9.53E-01	0.654075	0.690921	
2368.31	2.37E-02	0.304451	0.770987	
2377.29	5.05E-01	0.220647	0.045842	
2391.19	9.48E-01	0.631463	0.441035	
2402.28	3.08E-02	0.286833	0.983017	
2408.23	5.78E-01	0.327281	0.613692	
2413.4	5.31E-02	0.482397	0.571283	
2426.1	3.66E-01	0.02499	0.316634	
2434.15	7.17E-01	0.108214	0.693643	
2447.17	1.10E-01	0.942837	0.017912	
2461.79	5.82E-02	0.703161	0.654385	
2468.59	4.93E-02	0.569941	0.812185	
2477.24	6.69E-01	0.616827	0.734535	
2489.85	4.18E-03	0.576596	0.253833	
2504.33	8.17E-01	0.684066	0.454381	
2522.29	1.44E-01	0.983347	0.854004	
2534.48	5.63E-03	0.541321	0.720525	
2541.38	5.34E-01	0.11588	0.889986	
2557.61	1.41E-01	0.328503	0.737752	
2574.7	6.04E-02	0.742481	0.848244	
2575.23	3.61E-01	0.992883	0.060379	
2582.72	5.47E-02	0.405301	0.96732	
2589.42	3.92E-01	0.523518	0.5907	
2606.34	5.14E-01	0.600535	0.534712	
2618.39	4.50E-01	0.77526	0.978752	
2633.93	5.55E-01	0.613549	0.826384	
2651.54	2.08E-02	0.495361	0.415946	
2662.39	6.74E-01	0.908718	0.115955	
2670.22	8.18E-02	0.450936	0.824596	
2676.55	1.82E-02	0.606734	0.32286	
2679.84	1.48E-01	0.752101	0.932497	
2706.25	6.46E-01	0.86368	0.820086	
2719.5	7.50E-02	0.789843	0.761859	
2736.37	2.89E-01	0.613014	0.213346	
2741.49	9.75E-02	0.354795	0.979227	
2751.74	1.30E-02	0.958517	0.32476	
2757.91	3.25E-02	0.617156	0.499462	
2764.59	8.09E-01	0.561965	0.090265	
2773.35	2.50E-01	0.523971	0.694248	
2781.34	7.61E-03	0.363251	0.424709	
2791.42	5.32E-02	0.852828	0.944314	
2816.03	1.61E-01	0.814955	0.540482	
2819.41	1.57E-01	0.446749	0.615896	
2837.36	1.27E-03	0.79377	0.278348	
2846.49	5.63E-01	0.280769	0.829736	
2858.51	3.74E-01	0.745443	0.640759	
2866.37	3.04E-02	0.238604	0.58655	
2871.87	1.17E-01	0.169615	0.281691	
2886.05	7.47E-02	0.291295	0.749355	
2887.36	3.33E-01	0.682197	0.758862	
2898.42	5.72E-02	0.910112	0.421391	
2903.69	1.89E-01	0.854335	0.603954	
2931.97	1.15E-01	0.932759	0.929897	
2932.5	5.40E-02	0.559205	0.492669	
2950.54	7.17E-01	0.962259	0.118461	
2960.39	3.30E-01	0.298974	0.689899	
2980.65	4.28E-01	0.958129	0.073463	
2994.83	1.97E-02	0.409879	0.46138	
3006.62	7.80E-01	0.551831	0.909798	
3021.4	3.29E-04	0.931392	0.390467	
3034.59	1.97E-03	0.918576	0.51626	
3051.6	2.44E-01	0.904975	0.956784	
3066.5	7.16E-02	0.818907	0.957061	
3075.76	8.70E-01	0.8399	0.2046	
3092.57	1.12E-02	0.080639	0.255383	
3100.61	1.64E-01	0.638837	0.242148	
3108.67	1.73E-01	0.914193	0.002161	
3122.78	3.23E-01	0.720844	0.57229	
3123.54	1.86E-01	0.529192	0.537151	
3133.65	7.83E-03	0.215321	0.3933	
3149.6	4.10E-02	0.493062	0.330851	
3165.84	4.81E-01	0.441993	0.678708	
3181.11	1.29E-01	0.341001	0.797797	
3191.56	3.17E-05	0.153908	0.472455	
3195.64	4.03E-02	0.64711	0.726989	
3207.66	9.31E-01	0.811466	0.930409	
3214.57	7.54E-02	0.539015	0.493758	
3222.77	5.13E-01	0.133349	0.035963	
3230.72	8.92E-02	0.426207	0.676447	
3237.91	1.45E-01	0.505178	0.313794	
3245.88	2.34E-01	0.550643	0.673447	
3253.61	1.59E-01	0.20421	0.544415	
3262.63	5.54E-02	0.605072	0.653259	
3273.74	3.76E-01	0.878006	0.675416	
3286.67	5.26E-01	0.78733	0.95843	
3297.55	1.71E-01	0.938195	0.615492	
3308.72	1.67E-01	0.889125	0.974328	
3313.4	1.01E-02	0.467279	0.97733	
3319.38	4.47E-02	0.715037	0.915365	
3329.8	7.47E-02	0.929542	0.725981	
3336.05	1.64E-02	0.884855	0.455197	
3350.68	5.60E-01	0.656848	0.25207	
3361.93	1.43E-02	0.855801	0.805505	
3366.65	1.92E-02	0.379384	0.420525	
3377.66	2.98E-03	0.885214	0.881353	
3384.75	6.76E-01	0.692239	0.413982	
3394.48	6.92E-02	0.63973	0.630956	
3408.71	1.82E-01	0.665102	0.71543	
3420.85	2.04E-02	0.987827	0.796614	
3435.66	7.06E-05	0.222183	0.443634	
3438.84	9.63E-02	0.299702	0.219182	
3449.71	5.95E-01	0.289476	0.134325	
3462.72	1.69E-01	0.522368	0.143018	
3478.95	4.78E-01	0.8633	0.19921	
3480.01	6.29E-01	0.608521	0.630027	
3500.83	5.55E-01	0.805945	0.204029	
3512.86	4.39E-01	0.918283	0.24856	
3520.9	1.09E-01	0.705156	0.470414	
3534.85	9.91E-03	0.806227	0.26075	
3548.83	7.97E-01	0.485551	0.49279	
3565.8	8.05E-01	0.240997	0.539643	
3582.81	8.92E-01	0.272105	0.105656	
3587.95	1.44E-01	0.451251	0.295106	
3597.97	5.36E-01	0.125452	0.939971	
3601.22	9.70E-01	0.266986	0.788125	
3613.97	1.76E-01	0.764413	0.125708	
3616.96	4.74E-01	0.185313	0.260317	
3634.91	7.97E-01	0.568082	0.709593	
3637.63	8.89E-01	0.912516	0.450373	
3649.08	6.32E-01	0.573797	0.110513	
3652.33	3.81E-01	0.293911	0.889681	
3663.1	3.48E-01	0.984729	0.338457	
3683.93	4.16E-03	0.524931	0.486316	
3698.77	5.40E-01	0.990761	0.335283	
3714.15	1.11E-03	0.202902	0.511816	
3716.92	1.83E-01	0.844844	0.802611	
3730.82	8.09E-01	0.114251	0.154822	
3739.93	1.45E-01	0.397434	0.119318	
3751.84	6.66E-01	0.811022	0.102468	
3774.95	8.96E-02	0.183165	0.167367	
3782.84	7.31E-01	0.363872	0.012972	
3799.92	7.15E-02	0.921892	0.028824	
3815.76	3.41E-01	0.907569	0.342232	
3837.28	3.50E-01	0.078878	0.349702	
3852.17	2.89E-01	0.220977	0.527122	
3869.15	2.25E-01	0.997621	0.0402	
3885.13	1.68E-01	0.632689	0.023682	
3887.45	2.86E-03	0.530665	0.296176	
3901.15	2.46E-03	0.539656	0.203023	
3913.31	8.18E-01	0.32521	0.256624	
3928.87	5.31E-01	0.600336	0.353866	
3935.1	8.82E-02	0.354593	0.271467	
3949.66	1.24E-01	0.704787	0.979138	
3951.22	2.01E-01	0.926463	0.646163	
3961.37	1.27E-01	0.430299	0.327688	
3978.85	4.27E-01	0.676723	0.756548	
3979.9	1.35E-01	0.958639	0.70757	
3992.18	8.60E-01	0.766854	0.052827	
4006.32	2.15E-03	0.683373	0.122351	
4007.11	4.56E-04	0.597012	0.166145	
4016.55	2.92E-02	0.459741	0.644381	
4025.21	5.36E-01	0.818053	0.487395	
4034.15	4.79E-01	0.70041	0.298388	
4036.25	6.97E-01	0.208663	0.919193	
4048.36	1.13E-01	0.880641	0.035134	
4064.18	4.31E-01	0.410657	0.247586	
4073.43	4.22E-01	0.775783	0.164071	
4087.44	9.06E-03	0.363684	0.368484	
4103.61	9.45E-01	0.563073	0.112846	
4121.4	8.17E-01	0.822778	0.087332	
4135.23	2.09E-02	0.379576	0.352159	
4151.49	6.66E-01	0.229025	0.41924	
4167.24	7.96E-01	0.096769	0.604659	
4170.18	5.71E-01	0.01441	0.110268	
4189.46	5.30E-01	0.044716	0.289354	
4198.04	7.87E-01	0.147583	0.729155	
4207.16	1.81E-01	0.537741	0.33681	
4222.21	8.77E-02	0.015211	0.405463	
4236.21	1.35E-02	0.132364	0.613206	
4255.37	4.24E-01	0.724382	0.790037	
4265.37	1.23E-01	0.39665	0.095624	
4281.34	6.44E-02	0.155734	0.427168	
4293.27	3.53E-01	0.482135	0.130543	
4306.57	1.38E-02	0.15888	0.546194	
4317.17	2.69E-03	0.488722	0.022443	
4319.08	1.80E-04	0.222835	0.016652	
4331.33	4.33E-01	0.722945	0.366098	
4354.52	8.59E-03	0.972076	0.797552	
4370.38	1.81E-01	0.685139	0.789778	
4386.27	4.85E-02	0.173622	0.816262	
4394.23	3.82E-02	0.760086	0.800042	
4410.71	1.56E-04	0.793821	0.315861	
4451.65	3.17E-02	0.675772	0.730465	
4526.3	8.51E-01	0.310832	0.081012	
4540.49	4.85E-03	0.460622	0.181239	
4550.39	2.97E-01	0.234569	0.611093	
4568.43	1.45E-03	0.43901	0.28912	
4583.45	5.95E-02	0.359463	0.424178	
4589.57	8.96E-01	0.845854	0.391309	
4595.69	7.08E-01	0.247322	0.570566	
4597.48	6.90E-01	0.726537	0.142576	
4611.02	1.42E-01	0.868392	0.61944	
4627.9	2.86E-03	0.409763	0.441044	
4646.61	1.75E-02	0.685726	0.116984	
4674.62	1.99E-01	0.184649	0.488611	
4696	9.41E-01	0.983331	0.89655	
4696.78	7.74E-01	0.585194	0.961855	
4713.56	8.51E-01	0.86953	0.678901	
4713.82	4.70E-01	0.907201	0.936416	
4735.03	2.47E-01	0.298343	0.419975	
4751.62	4.88E-01	0.783279	0.669005	
4751.88	6.62E-01	0.571168	0.965441	
4757.07	7.93E-02	0.495335	0.866859	
4775.26	4.53E-02	0.530992	0.734864	
4793.75	2.13E-01	0.441831	0.404697	
4806.79	5.49E-02	0.359093	0.303788	
4818.28	1.33E-02	0.340289	0.493028	
4850.46	8.87E-02	0.657265	0.430134	
4863.58	2.02E-01	0.513871	0.683576	
4889.87	3.76E-01	0.565365	0.416742	
4905.41	8.67E-01	0.859776	0.571543	
4916.49	2.76E-01	0.994948	0.890922	
4928.63	4.72E-01	0.663554	0.175905	
4953.24	1.90E-01	0.65716	0.408792	
5007.16	2.27E-02	0.475184	0.489004	
5059.77	6.10E-01	0.85691	0.206575	
5079.33	2.62E-02	0.232057	0.434911	
5093.56	2.46E-01	0.548188	0.7091	
5171.49	1.12E-01	0.079004	0.849667	
5186.38	7.50E-01	0.075594	0.96761	
5216.79	4.61E-01	0.077071	0.310989	
5233.39	1.66E-01	0.199764	0.29503	
5249.74	4.75E-01	0.27476	0.467136	
5268.84	6.28E-01	0.631955	0.097808	
5284.7	4.68E-01	0.435144	0.231524	
5300.86	6.89E-01	0.268444	0.414986	
5314.57	5.52E-01	0.054767	0.260813	
5329.67	4.87E-01	0.036384	0.166868	
5362.42	2.96E-01	0.219846	0.422679	
5377.87	8.42E-01	0.125616	0.400654	
5386.98	2.01E-01	0.25758	0.540398	
5403.57	3.33E-01	0.555077	0.49085	
5417.96	7.25E-01	0.854116	0.925816	
5445.7	3.65E-03	0.22211	0.418435	
5469.09	1.43E-02	0.566547	0.735026	
5471.85	6.16E-01	0.917511	0.565267	
5495.93	5.41E-01	0.305397	0.334268	
5497.69	2.47E-01	0.474342	0.391339	
5512.01	5.94E-01	0.895494	0.497027	
5515.03	4.74E-02	0.210641	0.933914	
5532.91	9.37E-01	0.439855	0.788917	
5566.98	1.97E-01	0.376623	0.723062	
5580.89	7.78E-01	0.796954	0.064149	
5590.51	9.77E-01	0.236007	0.225161	
5593.04	4.59E-01	0.449518	0.336541	
5620.69	6.22E-01	0.738847	0.885226	
5640.01	1.90E-02	0.725177	0.728044	
5662.93	1.72E-01	0.399795	0.356736	
5676.96	6.09E-01	0.129099	0.386197	
5700.46	3.30E-03	0.983486	0.314051	
5715.82	4.56E-01	0.678617	0.92356	
5767.93	2.96E-01	0.858337	0.510308	
5784.15	9.65E-01	0.205127	0.958405	
5803.48	9.35E-02	0.856352	0.409128	
5847.18	2.65E-02	0.748206	0.359653	
5860.14	6.28E-01	0.437919	0.202247	
5870	4.68E-01	0.529237	0.811534	
5884.54	4.42E-02	0.051456	0.386013	
5914.46	3.80E-02	0.06249	0.271825	
5931.15	5.16E-01	0.770411	0.233173	
5945.77	5.21E-01	0.946183	0.436674	
5963.02	9.46E-01	0.47428	0.94537	
5982.65	6.64E-02	0.977926	0.716633	
5998.65	8.52E-01	0.28249	0.226388	
6021.49	8.43E-02	0.142234	0.545759	
6032.8	2.63E-01	0.047042	0.796996	
6057.3	6.91E-01	0.654144	0.937607	
6087.39	9.32E-01	0.37515	0.353062	
6098.5	5.08E-01	0.868513	0.953607	
6113.32	5.41E-02	0.93535	0.613575	
6152.85	3.40E-01	0.165956	0.944665	
6176.52	5.68E-03	0.052457	0.770967	
6199.97	2.49E-01	0.374315	0.402658	
6215.72	5.73E-01	0.246853	0.865282	
6239.24	6.73E-01	0.657878	0.733805	
6251.83	4.17E-01	0.339993	0.272373	
6306.59	3.01E-01	0.997046	0.938315	
6322.74	7.35E-01	0.135118	0.7215	
6333.25	7.87E-02	0.164924	0.17371	
6339.18	1.30E-01	0.913244	0.378552	
6368.07	4.76E-01	0.172556	0.261893	
6385.92	7.11E-01	0.546485	0.858749	
6401.91	6.90E-01	0.679013	0.614104	
6416.82	3.37E-01	0.312064	0.685413	
6433.39	6.20E-02	0.310916	0.624734	
6449.97	2.19E-01	0.753195	0.905696	
6485.11	6.70E-02	0.273606	0.556946	
6515.16	8.66E-01	0.516697	0.384065	
6531.57	6.29E-01	0.289491	0.226678	
6562	8.83E-01	0.80236	0.522714	
6564.47	2.48E-01	0.463092	0.657313	
6580.4	6.39E-01	0.442607	0.17436	
6597.17	8.60E-01	0.904277	0.907274	
6614.24	5.90E-01	0.407152	0.656052	
6629.4	8.63E-01	0.783434	0.377739	
6645.96	8.67E-01	0.863144	0.650047	
6661.99	4.29E-01	0.359843	0.611694	
6711.57	1.04E-01	0.855689	0.201736	
6726.57	2.56E-02	0.917614	0.76796	
6743.25	4.89E-01	0.491839	0.330206	
6759.39	6.98E-01	0.646858	0.258447	
6776.39	1.75E-02	0.893875	0.565599	
6816.61	7.09E-02	0.905187	0.88832	
6845.44	7.57E-01	0.797322	0.932749	
6860.59	7.15E-01	0.319522	0.950628	
6875.75	1.79E-01	0.869848	0.269782	
6892.33	9.41E-01	0.550186	0.396864	
6896.95	6.53E-01	0.809593	0.393583	
6915.72	9.67E-01	0.959927	0.63942	
6931.76	1.01E-01	0.637204	0.411977	
6942.29	5.20E-01	0.151546	0.224241	
6954.09	3.53E-01	0.211203	0.223169	
7001.12	8.89E-01	0.868468	0.573189	
7005.66	9.85E-01	0.190528	0.909438	
7018.52	3.84E-01	0.267038	0.719638	
7021.29	7.82E-01	0.141469	0.457908	
7044.53	8.69E-01	0.174971	0.836883	
7063.24	7.87E-01	0.946735	0.862539	
7063.75	4.42E-01	0.996558	0.897565	
7064.76	4.94E-01	0.835132	0.758884	
7088.82	4.15E-02	0.452482	0.964286	
7112.17	9.40E-01	0.688314	0.937033	
7118.77	9.06E-01	0.043705	0.496007	
7136.57	2.79E-01	0.945137	0.723321	
7161.26	1.26E-02	0.561482	0.741105	
7201.07	8.34E-02	0.347025	0.771061	
7221.78	2.21E-02	0.178024	0.608825	
7237.14	1.39E-02	0.220996	0.558483	
7260.73	3.32E-02	0.922165	0.952971	
7289.75	5.05E-02	0.32271	0.301	
7308.02	8.13E-01	0.077894	0.718857	
7326.05	6.87E-03	0.795931	0.958653	
7341.26	4.25E-02	0.667326	0.965875	
7356.23	1.34E-01	0.330106	0.744017	
7372.77	4.88E-02	0.810664	0.846803	
7390.88	7.72E-04	0.673241	0.674631	
7429.5	2.00E-01	0.66923	0.63917	
7453.14	5.84E-02	0.478179	0.513474	
7561.94	4.47E-02	0.328069	0.867249	
7573.99	1.29E-02	0.282399	0.883725	
7616.76	1.15E-01	0.572301	0.530802	
7841.24	3.43E-04	0.36888	0.492042	
7857.51	5.53E-03	0.94971	0.211348	
7923.31	2.94E-03	0.865775	0.263475	
7923.85	1.25E-01	0.87817	0.269608	
8076.02	9.66E-01	0.186915	0.886203	
8182.44	2.53E-02	0.517342	0.603309	
8190.61	3.33E-02	0.012797	0.624357	
8255.87	1.61E-01	0.14599	0.742615	
8270.11	8.50E-02	0.746648	0.863047	
8294.77	1.99E-02	0.735959	0.903825	
8410.08	5.21E-03	0.385973	0.367718	
8933.53	2.04E-02	0.484712	0.486734	
8950.05	2.14E-01	0.598509	0.660873	
8950.33	1.65E-02	0.548966	0.581307	
8999.68	2.61E-03	0.501968	0.369918	
9003.11	5.67E-02	0.877714	0.541587	
9022.23	1.68E-02	0.313905	0.461584	
9065.33	4.20E-04	0.177063	0.245717	
9089.43	1.09E-04	0.202433	0.12991	
9106.77	1.88E-03	0.174218	0.18486	
9123.87	5.41E-05	0.184106	0.382374	
9166.96	4.91E-02	0.131293	0.264319	
9184.37	2.81E-04	0.336984	0.585751	
10029.4	1.46E-02	0.498838	0.242658	
10592	1.95E-04	0.175873	0.192254	
11138.5	3.55E-02	0.687364	0.715145	
11251.4	9.07E-03	0.180932	0.700183	
11608.6	4.09E-04	0.624184	0.996229	
12244.5	2.32E-02	0.153197	0.891545	
12274.5	1.02E-01	0.067447	0.197798	
12832.9	3.48E-04	0.268696	0.359724	
12880.5	2.57E-03	0.47783	0.846144	
12986.3	5.66E-04	0.279846	0.329795	
14019.8	2.22E-01	0.777149	0.964325	
14081.9	5.42E-01	0.875479	0.90564	
14125.1	2.61E-01	0.92566	0.933614	
14126.7	2.65E-01	0.954641	0.90496	
14127.3	4.49E-01	0.890792	0.466541	
14174.3	3.61E-01	0.807694	0.875049	
14224.6	8.37E-01	0.511285	0.543366	
14224.9	9.84E-01	0.442034	0.566658	
14271.8	1.16E-01	0.825192	0.447609	
14272.1	8.85E-01	0.687884	0.845414	
14324.4	9.53E-03	0.687187	0.746382	
14371.5	8.84E-04	0.632339	0.817311	
14520.5	9.83E-02	0.934121	0.554036	
14679.6	8.49E-02	0.700226	0.389169	
14695.2	9.23E-01	0.506784	0.149577	
14715.8	6.91E-02	0.703766	0.470123	
14716.9	7.59E-01	0.588645	0.874335	
14730.8	6.26E-01	0.807362	0.933284	
15018.1	4.51E-02	0.9114	0.749495	
15102.1	5.21E-02	0.792467	0.88305	
15783.3	6.20E-03	0.433038	0.639794	
15845.3	4.67E-03	0.451027	0.113162	
15846.4	5.51E-02	0.623402	0.396027	
17626.7	1.75E-02	0.567451	0.738612	
18329.2	2.30E-01	0.943404	0.768311	
18454	6.45E-01	0.537913	0.718472	
18909.3	1.23E-01	0.708919	0.751594	
18951	2.36E-01	0.567866	0.540165	
