# Supplementary material for: A Metaproteomic Approach to Study Human-Microbial Ecosystems at the Mucosal Luminal Interface
Source: PLoS One. 2011 Nov 21;6(11):e26542. doi: 10.1371/journal.pone.0026542 (PMC3221670; doi:10.1371/journal.pone.0026542)
Supplement: Table S4 — P-values of 49 proteins with biogeographic feature. (RTF) [file pone.0026542.s004.rtf]

 Table S4. P-values of 49 proteins with biogeographic feature
ANOVA of 4 regions	ANOVA of 2 regions	Protein ID	Description	
0.110	0.025	AMYP	Pancreatic alpha-amylase OS=Homo sapiens GN=AMY2A PE=1 SV=2	
0.112	0.088	CEL3A	Chymotrypsin-like elastase family member 3A OS=Homo sapiens GN=CELA3A PE=1 SV=3	
0.621	0.371	IGKC	Ig kappa chain C region OS=Homo sapiens GN=IGKC PE=1 SV=1	
0.138	0.240	FCGBP	IgGFc-binding protein OS=Homo sapiens GN=FCGBP PE=1 SV=3	
0.000	0.000	IGHA2	Ig alpha-2 chain C region OS=Homo sapiens GN=IGHA2 PE=1 SV=3	
0.166	0.950	A1AT	Alpha-1-antitrypsin OS=Homo sapiens GN=SERPINA1 PE=1 SV=3	
0.070	0.286	CEL2A	Chymotrypsin-like elastase family member 2A OS=Homo sapiens GN=CELA2A PE=1 SV=1	
0.699	0.980	CBPB1	Carboxypeptidase B OS=Homo sapiens GN=CPB1 PE=1 SV=4	
0.365	0.504	IGHM	Ig mu chain C region OS=Homo sapiens GN=IGHM PE=1 SV=3	
0.092	0.398	CBPA1	Carboxypeptidase A1 OS=Homo sapiens GN=CPA1 PE=1 SV=2	
0.018	0.414	MGA	Maltase-glucoamylase, intestinal OS=Homo sapiens GN=MGAM PE=1 SV=5	
0.194	0.322	CLCA1	Calcium-activated chloride channel regulator 1 OS=Homo sapiens GN=CLCA1 PE=1 SV=2	
0.366	0.106	PPBI	Intestinal-type alkaline phosphatase OS=Homo sapiens GN=ALPI PE=1 SV=2	
0.489	0.144	LAC	Ig lambda chain C regions OS=Homo sapiens GN=IGLC1 PE=1 SV=1	
0.804	0.727	TRY2	Trypsin-2 OS=Homo sapiens GN=PRSS2 PE=1 SV=1	
0.420	0.285	HV305	Ig heavy chain V-III region BRO OS=Homo sapiens PE=1 SV=1	
0.003	0.002	PIGR	Polymeric immunoglobulin receptor OS=Homo sapiens GN=PIGR PE=1 SV=4	
0.316	0.248	CTRC	Chymotrypsin-C OS=Homo sapiens GN=CTRC PE=1 SV=2	
0.017	0.003	CEL3B	Chymotrypsin-like elastase family member 3B OS=Homo sapiens GN=CELA3B PE=1 SV=2	
0.157	0.032	IGHA1	Ig alpha-1 chain C region OS=Homo sapiens GN=IGHA1 PE=1 SV=2	
0.045	0.379	SUIS	Sucrase-isomaltase, intestinal OS=Homo sapiens GN=SI PE=1 SV=5	
0.527	0.982	DMBT1	Deleted in malignant brain tumors 1 protein OS=Homo sapiens GN=DMBT1 PE=1 SV=2	
0.087	0.014	ENPP7	Ectonucleotide pyrophosphatase/phosphodiesterase family member 7 OS=Homo sapiens GN=ENPP7 PE=1 SV=3	
0.069	0.044	MEP1A	Meprin A subunit alpha OS=Homo sapiens GN=MEP1A PE=2 SV=2	
0.072	0.332	SBP1	Selenium-binding protein 1 OS=Homo sapiens GN=SELENBP1 PE=1 SV=2	
0.120	0.694	ASAH2	Neutral ceramidase OS=Homo sapiens GN=ASAH2 PE=1 SV=2	
0.279	0.288	LPH	Lactase-phlorizin hydrolase OS=Homo sapiens GN=LCT PE=1 SV=2	
0.262	0.904	PCD24	Protocadherin-24 OS=Homo sapiens GN=PCDH24 PE=1 SV=2	
0.528	0.339	SPB6	Serpin B6 OS=Homo sapiens GN=SERPINB6 PE=1 SV=3	
0.224	0.098	AMY2B	Alpha-amylase 2B OS=Homo sapiens GN=AMY2B PE=1 SV=1	
0.089	0.453	MEP1B	Meprin A subunit beta OS=Homo sapiens GN=MEP1B PE=1 SV=2	
0.487	0.243	ACE	Angiotensin-converting enzyme OS=Homo sapiens GN=ACE PE=1 SV=1	
0.575	0.370	K2C1	Keratin, type II cytoskeletal 1 OS=Homo sapiens GN=KRT1 PE=1 SV=6	
0.002	0.051	ENTK	Enteropeptidase OS=Homo sapiens GN=PRSS7 PE=2 SV=2	
0.001	0.000	CEAM5	Carcinoembryonic antigen-related cell adhesion molecule 5 OS=Homo sapiens GN=CEACAM5 PE=1 SV=2	
0.967	0.764	HV303	Ig heavy chain V-III region VH26 OS=Homo sapiens PE=1 SV=1	
0.504	0.330	AACT	Alpha-1-antichymotrypsin OS=Homo sapiens GN=SERPINA3 PE=1 SV=2	
0.017	0.080	ILEU	Leukocyte elastase inhibitor OS=Homo sapiens GN=SERPINB1 PE=1 SV=1	
0.107	0.028	DPEP1	Dipeptidase 1 OS=Homo sapiens GN=DPEP1 PE=1 SV=3	
0.111	0.374	DPP4	Dipeptidyl peptidase 4 OS=Homo sapiens GN=DPP4 PE=1 SV=2	
0.306	0.050	KV205	Ig kappa chain V-II region GM607 (Fragment) OS=Homo sapiens PE=4 SV=1	
0.128	0.015	IGJ	Immunoglobulin J chain OS=Homo sapiens GN=IGJ PE=1 SV=4	
0.702	0.373	G3P	Glyceraldehyde-3-phosphate dehydrogenase OS=Homo sapiens GN=GAPDH PE=1 SV=3	
0.661	0.419	SODC	Superoxide dismutase [Cu-Zn] OS=Homo sapiens GN=SOD1 PE=1 SV=2	
0.035	0.002	IGHG1	Ig gamma-1 chain C region OS=Homo sapiens GN=IGHG1 PE=1 SV=1	
0.217	0.257	MUCDL	Mucin and cadherin-like protein OS=Homo sapiens GN=MUPCDH PE=1 SV=2	
0.252	0.362	MUC2	Mucin-2 OS=Homo sapiens GN=MUC2 PE=1 SV=2	
0.919	0.549	HV320	Ig heavy chain V-III region GAL OS=Homo sapiens PE=1 SV=1	
0.000	0.000	KV309	Ig kappa chain V-III region VG (Fragment) OS=Homo sapiens PE=1 SV=1	
